# Stable isotopic characterization of a coastal floodplain forest community: a case study for isotopic reconstruction of Mesozoic vertebrate assemblages

**DOI:** 10.1098/rsos.181210

**Published:** 2019-02-20

**Authors:** T. M. Cullen, F. J. Longstaffe, U. G. Wortmann, M. B. Goodwin, L. Huang, D. C. Evans

**Affiliations:** 1 Department of Ecology and Evolutionary Biology, University of Toronto, 25 Willcocks Street, Toronto, Ontario, Canada M5S 3B2; 2 Department of Earth Sciences, The University of Western Ontario, 1151 Richmond Street, London, Ontario, Canada N6A 5B7; 3 Department of Earth Sciences, University of Toronto, 22 Russell Street, Toronto, Ontario, Canada M5S 3B1; 4 University of California Museum of Paleontology, 1101 Valley Life Sciences, Berkeley, CA 94720-4780, USA; 5 Royal Ontario Museum, 100 Queen's Park, Toronto, Ontario, Canada M5S 2C6

**Keywords:** stable isotope, palaeoecology, community ecology, ecosystem, Mesozoic, analogue

## Abstract

Stable isotopes are powerful tools for elucidating ecological trends in extant vertebrate communities, though their application to Mesozoic ecosystems is complicated by a lack of extant isotope data from comparable environments/ecosystems (e.g. coastal floodplain forest environments, lacking significant C_4_ plant components). We sampled 20 taxa across a broad phylogenetic, body size, and physiological scope from the Atchafalaya River Basin of Louisiana as an environmental analogue to the Late Cretaceous coastal floodplains of North America. Samples were analysed for stable carbon, oxygen and nitrogen isotope compositions from bioapatite and keratin tissues to test the degree of ecological resolution that can be determined in a system with similar environmental conditions, and using similar constraints, as those in many Mesozoic assemblages. Isotopic results suggest a broad overlap in resource use among taxa and considerable terrestrial–aquatic interchange, highlighting the challenges of ecological interpretation in C_3_ systems, particularly when lacking observational data for comparison. We also propose a modified oxygen isotope-temperature equation that uses mean endotherm and mean ectotherm isotope data to more precisely predict temperature when compared with measured Atchafalaya River water data. These results provide a critical isotopic baseline for coastal floodplain forests, and act as a framework for future studies of Mesozoic palaeoecology.

## 1. Introduction

Naturally existing stable isotopes in the environment are taken up by organisms through their feeding and drinking behaviour, and incorporated into their tissues in ways that reflect their diet, habitat, physiology and food web structure [1–10]. Stable carbon, nitrogen and oxygen isotopes, in particular, can be analysed to provide critical information about diet, habitat preference, trophic structure, physiology and temperature. Soft tissues (e.g. blood, muscle, collagen, keratin) are frequently used in stable isotope studies of living organisms, as they record ecologically meaningful signals over small periods of time (typically days or months), can be harvested from live specimens with minimal undue harm, and typically have small trophic enrichment factors relative to diet [1,4,8,11–14]. Additionally, because these tissues are more easily and repeatedly harvested, they are also well suited to controlled diet experiments to determine trophic enrichment factors (TEFs) (differences between the isotopic composition of diet and resulting tissues once incorporated into an organism). By contrast, most palaeobiological applications of stable isotope analysis rely on the use of hard tissues (e.g. bone, teeth or scales) as soft tissues are generally not thought to be preserved in ways that are conducive to deep-time stable isotope studies [4,15–35]. As a result, stable isotope studies of most fossils older than the Pleistocene, and particularly fossils from Mesozoic or older systems, are typically focused on bioapatite (i.e. hydroxyapatite, [Ca_5_(PO_4_,CO_3_)_3_(OH,CO_3_)]) in hard tissues such as bones and teeth [4,20,21,34,35]. Given the relative paucity of available comparative stable isotope ecological and experimental diet fractionation data from these hard tissues for many taxa in modern systems, and the lack of more trophically informative stable isotopes such as nitrogen when analysing inorganic tissue components, formulating and testing predictions about ancient ecological communities using stable isotope analysis can be particularly challenging [4,5,20,33,34,36–39].

The most significant obstacle when using stable isotope methods in deep time contexts is that the extant ecosystems are not necessarily reflective of those that most commonly existed during much of Earth history, such as in Mesozoic systems [5,20,34,40]. These ancient systems existed during periods that were typically warmer and wetter than many contemporary environments [25,35,41–44], their vertebrate communities were typically reptile-dominated [45–47], there was little to no C_4_ component [21,34,42], and many often had some degree of marine input [43,45–47]. As many community-level isotopic analyses today focus on mammal-dominated systems [1,3,7,17,48–54], and/or temperate [15,16,50–53,55,56] or arid environments [1,3,5,57–63], direct comparisons to ancient coastal floodplain communities require careful consideration. Furthermore, non-mammalian taxa themselves are studied less frequently and are often the subject of more specific investigations such as migration (e.g. birds) or environmental toxins (e.g. amphibians) rather than more general analyses of isotopic variation, diet, habit use and/or community ecology that would be of great interest to evolutionary ecologists and palaeobiologists [4,8,26,64–69].

There is a growing interest in applying isotopic approaches to ecological questions in deep time contexts [21,23,24,28,34,35,70]; however, these studies suffer from the lack of comparative data on C_3_ fluviodeltaic and coastal ecosystems on which to base their ecological interpretations. Here, we provide the first comprehensive framework for assessing isotope variation in terrestrial and aquatic vertebrate communities within a C_3_-dominated subtropical coastal floodplain forest system with considerable terrestrial–aquatic interchange. We address the issues stated above through an intensive sampling of multiple tissues from a wide array of vertebrate taxa (including mammals, reptiles and fish) from a single community, the Atchafalaya River Basin (ARB) in Louisiana [71,72], and by assessing tissue and taxon level variability in carbon, oxygen and nitrogen isotopes in these samples. Since these ecosystems are common in the fossil record, this study will not only enhances our understanding of isotopic variation in extant vertebrate food webs, but will facilitate ecological comparisons using isotopes in deep time.

## 2. Material and methods

### 2.1. Experimental scope and specific objectives

By sampling both soft and hard tissues of taxa with a wide phylogenetic scope, range of body sizes, and physiological range (ectotherms and endotherms), and comparing the resultant isotopic data with both observational natural history data and previously collected isotopic discrimination and environmental data, we intend to fill in gaps in existing knowledge, and facilitate greater connectivity between stable isotope analyses in modern and ancient settings. The specific objectives of this research are to: (i) analyse the community ecology and isotopic resource use of various taxa in the Atchafalaya system using stable carbon, oxygen and nitrogen isotopes; (ii) test the hypotheses that individual species isotopic ranges are distinct and will not show considerable overlap, thereby allowing relative determination of trophic niches; (iii) demonstrate that temperature estimates from species oxygen isotope concentrations will correspond with local measured water temperature data; and (iv) determine if relative positions of species in isotopic space reflect partitioning of resources and micro-habitat preferences that can used for inference in future studies of Mesozoic ecosystems.

### 2.2. Specimen collection

**Figure 1.**
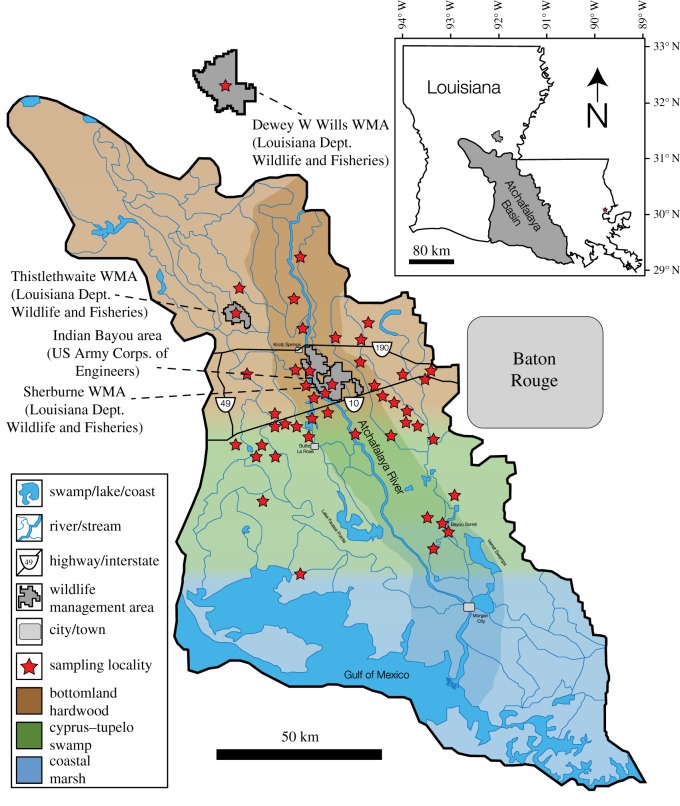
Sampling map of Atchafalaya River Basin, Louisiana. Stars represent locations of collected samples. Although some localities only correspond to a single specimen, many correspond to multiple specimens (see electronic supplementary material, table S1 for detailed specimen collection data).

Specimens were collected from the Atchafalaya River Basin (ARB), a region in southern Louisiana containing a range of environments, including bottomland hardwood forests of the lower Mississippi Alluvial Valley, vast wetland areas dominated by bald cypress (*Taxodium distichum*) and water tupelo (*Nyssa aquatica*), and coastal marshes [71]. Recent surveys have indicated that the ARB habitats are approximately 70% forest, with the remaining 30% wetland and open water settings, and that the ARB represents the largest contiguous expanse of coastal floodplain forest in the continental USA [71]. Samples were collected opportunistically by roadside survey by TMC and DCE over an approximately 70 km transect of the ARB in February and October 2015 (figure 1), in collaboration with the Louisiana Department of Wildlife and Fisheries (LDWF). For each specimen, multiple tissues were sampled when possible, with the goal of obtaining multiple keratin (hair and claw) and bioapatite (bone and tooth) tissues per specimen. Samples obtained during inspections and other monitoring programmes were provided by the LDWF. Additional samples were provided by University of Louisiana at Lafayette researcher Dr Jim Delahoussaye (various, but in particular fish and crocodilians), and Louisiana State University Museum of Natural Science (LSU-MNS) curator Dr Jacob Esselstyn (large carnivorous mammals). These are included with the sample locations/data noted in figure 1.

### 2.3. Sample selection

Specimens were selected from the total sample (electronic supplementary material, table S1) in order to represent as broad a range of taxa, tissues and ecological factors (i.e. body size, diet, physiology, habitat preference) as possible. Natural history data for sampled taxa were collected from the literature and compiled in electronic supplementary material, table S2. Owing to time and budgetary constraints, not all samples could be analysed. A total of 105 specimens in total were selected from 20 species, including fish such as *Amia calva* (bowfin, *N* = 4), *Lepisosteus sp*. (gar, *N* = 4) and *Atractosteus spatula* (alligator gar, *N* = 5), reptiles such as *Alligator mississippiensis* (American alligator, N = 6), a metatherian mammal *Didelphis virginiana* (Virginia opossum, *N* = 9) and eutherian mammals including *Sylvilagus aquaticus* (swamp rabbit, *N* = 4), *Odocoileus virginianus* (white-tailed deer, *N* = 30), *Sciurus niger* (fox squirrel, *N* = 6), *Peromyscus gossypinus* (cotton mouse, *N* = 1), *Neotoma floridana* (eastern woodrat, *N* = 1), *Myocastor coypus* (nutria, *N* = 2), *Dasypus novemcinctus* (nine-banded armadillo, *N* = 5), *Sus scrofa* (feral pig, *N* = 2), *Procyon lotor* (raccoon, *N* = 5), *Mephitis mephitis* (striped skunk, *N* = 3), *Neovison vison* (American mink, *N* = 2), *Lontra canadensis* (North American river otter, *N* = 3), *Canis latrans* (coyote, *N* = 6), *Lynx rufus* (bobcat, *N* = 3) and *Ursus americanus* (black bear, *N* = 4). *Lepisosteus* specimens could not be determined to species level at all times due to the nature of some of the collected material, and may represent either *L. osseus* (longnose gar) or *L. oculatus* (spotted gar), and are thus treated together.

### 2.4. Sample pre-treatment

Sample pre-treatments were performed on teeth and bones prior to stable isotope analyses. The purpose of these treatments was to reduce or eliminate contributions from organic matter and secondary carbonate to the carbon dioxide released by laser heating. For most samples, surfaces were manually cleaned, then sonicated in Millipore water for 15 min. Surfaces were coated in 2% NaOCl, and allowed to react for 24 h. Samples were then rinsed five times in Millipore water and dried for 24 h under vacuum at approximately 80°C and approximately 20–25 mmHg to remove remaining water and other sorbed volatiles, and kept under vacuum until laser ablation. A subset of teeth and bones used for laser isotopic analyses, as well as all samples powdered for bioapatite structural carbonate isotopic analyses, were given additional pre-treatment. This sample pre-treatment was adapted from Larsen & Longstaffe [73], with further secondary-carbonate removal modifications based on Snoeck & Pelligrini [74] and Pelligrini & Snoeck [75]. Samples were sonicated for 1 h in vials containing an excess of 2% NaOCl, then rinsed in Millipore water. An excess of 2% NaOCl was then added, with samples covered and left to react for 24 h. Samples were then rinsed five times in Millipore water, before an excess of 1 M acetic acid buffered with Na-acetate (or Ca-acetate, depending on availability) was added for 1 h, with sonication for the last 10 min. The samples were then rinsed five times in Millipore water, including 10 min sonication during the last rinse. The specimens were then lyophilized for 24 h.

Keratin pre-treatment methods were modified from O'Connell & Hedges [76] and O'Connell *et al*. [77]. Hair samples were cleaned to remove surface contaminants via soaking for 1 h in a 2 : 1 mixture of methanol and chloroform. Treated samples were then rinsed twice in deionized water and dried for 24 h. For claw keratin, manual surface cleaning was performed, followed by a 10 min rinse and sonication, and overnight drying at 60°C. Following this, claw keratin samples were cleaned following the hair keratin procedure described above.

### 2.5. Isotopic analysis

The ratio of heavy to light isotope is typically reported as parts per thousand (per mil, ‰) relative to a known standard (denoted using *δ*, where *δ* = [[Rsample/Rstandard]−1]) [1,4,8,20,78,79]. Certain stable isotopes, such as carbon (indicated as the ratio ^13^C/^12^C or as *δ*^13^C, reported hereafter relative to Vienna Pee-Dee Belemnite or VPDB), nitrogen (indicated as the ratio ^15^N/^14^N or as *δ*^15^N, reported hereafter relative to atmospheric N_2_ or AIR) and oxygen (indicated as the ratio ^18^O/^16^O or *δ*^18^O, reported hereafter relative to Vienna Standard Mean Ocean Water or VSMOW), have been extensively used to study the ecology of a wide variety of taxa [1,3,9,14,15,26,50–54,56,58–60,64–69,73,80–105] from a range of different habitats [1,17,18,49,52,54,57,106–110]. Which isotope is used depends on the study questions, as different isotopic ratios relate to different aspects of the ecology, physiology and environment of an organism [1,3,4,20,78,111].

Given the nature of the material and isotopes analysed for this project, several different analytical techniques were employed.

The laser-ablation gas-chromatography isotope-ratio-mass-spectrometry (LA-GC-IRMS) system used in this study is the same as that reported in Larson & Longstaffe [73], and is modified from the system described by Cerling & Sharp [112]. The system uses a 25 W New-Wave MIR10 CO_2_ gas source IR-laser with a wavelength of 10.66 mm, and the laser was set to produce 60 ms pulses, 180 mm diameter spots, and to fire using 15–20% power (dependent somewhat on sample material and reflectivity). Samples were loaded into a 3.8 cm diameter sample chamber with 20 cm^3^ volume designed for rapid sample switching, kept under a constant flow of approximately 40 ml min^−1^ of ultra-high purity helium, with CO_2_ gas liberated through ablation travelling to a Thermo Scientific^TD^ GasBench II, being cryogenically focused in a liquid nitrogen trap for 3 min, then heated to 25°C, passed through a Nafion water trap and then moved into a Thermo Scientific^TD^ Delta^plus^XL mass spectrometer for measurement. The sample chamber was heated and kept at approximately 50°C at all times, and background CO_2_ and H_2_O blanks were measured prior to each sample ablation session to ensure proper pre-analysis chamber and sample degassing. Calibrations were performed based on repeated measurements of NBS-18 and NBS-19, along with repeated analyses of a polished slab of an internal laboratory standard calcite (WS-1) to monitor/correct for analytical drift between sessions, as per [73].

Carbon and oxygen isotope compositions of bone bioapatite structural carbonate were analysed using a continuous-flow isotope-ratio-mass spectrometry system (CF-IRMS), beginning with weighed powders (approx. 1000 µg) being reacted with orthophosphoric acid to produce CO_2_ at 70°C for 60 min, then sampled through a Thermo Scientific^TD^ Gasbench II at a column temperature of 60°C, and then measured in continuous-flow mode, along with a CO_2_ reference gas, using a Thermo Scientific^TD^ MAT253 IRMS. Values of *δ*^13^C and *δ*^18^O were calculated based on calibrations performed on repeated measurements of IAEA-CO-8, NBS 19 and IAEA-CO-1 standards.

Carbon and nitrogen isotope compositions of hair and claw keratin were analysed using a continuous-flow isotope-ratio-mass-spectrometer system (CF-IRMS) via an automated Eurovector elemental analyser (EA) coupled to a Thermo Scientific MAT253 IRMS. Clipped keratin samples were weighed (approx. 200 µg) and placed into tin cups, and then dropped individually to the EA, where the keratin is converted to CO_2_ and NO_x_ (later reduced to N_2_) through flash combustion at approximately 1700°C under oxygen atmosphere conditions. *δ*^13^C and *δ*^15^N calibrations were determined through repeated measurements of IAEA-CH-3, IAEA-CO-1, USGS-34, IAEA-NO-3 and IAEA-N-2 standards.

For oxygen isotope analysis of claw and hair keratin samples, weighed keratin clippings (approx. 180 µg) were added to silver cups, then pyrolysed using an automated HEKAtech HT-EA oxygen analyser at approximately 1400°C using helium as a carrier gas. The CO gas produced through this process was passed through an Ascarite trap, separated on a 5 Å Molsieve, and then measured using a Thermo Scientific^TD^ MAT253 IRMS in continuous-flow mode using Conflo III open split interface. Calibrations for *δ*^18^O were based on repeated measurements of USGS-42 and USGS-43 keratin standards.

### 2.6. Trophic enrichment factors and oxygen isotope water calculations

Differences often exist between stable isotope compositions of carbon, nitrogen and oxygen as recorded in a sampled tissue, and the compositions of those same isotopes in the original diet or other intake for an organism [1,2,4,8]. This difference between the tissue signal and diet signal is controlled by a number of factors, including physiology, environment, dietary composition and/or the particular tissue being analysed. This is commonly referred to as the TEF (the trophic enrichment factor, trophic discrimination factor, diet fractionation factor, diet isotope offsets, fractionation factor, discrimination factor, isotopic separation, isotopic spacing, summed vital effects etc.) [1,2,4,8,12,15,29,49,81,86,89,94,110,111,113]. For simplicity, we denote TEFs and tissue–tissue isotopic differences as Δ, and calculated as Δ = *δ*_tissue_ − *δ*_diet_ (or *δ*_diet_ = *δ*_tissue_ − Δ) or Δ = *δ*_tissue_ − *δ*_tissue_ [1,2,4,8,15,29,86,111]. These factors are commonly specific to a given species, taxonomic group and/or dietary type (i.e. herbivore, faunivore), and can be empirically determined using controlled diet experiments [9,49,64–66,68–70,77,80,81,84,88,90,91,94–96,98–100,105]. Unfortunately, these experiments are often time-intensive and costly, and as such the dietary fractionations for each tissue are not known for most species [4,8,9,11,12], nor is the full range of variability in these factors or their relation to diet and physiology completely understood [11,12,114–116]. Despite these uncertainties, dietary fractionation factors for close relatives, or taxa with similar diets, are commonly used when a species-specific factor is unavailable [1,4,5,111], although more complex Bayesian approaches also exist for predicting TEFs [117]. Here, we use TEFs (Δ) obtained from the literature searches, and compiled in electronic supplementary material, table S3. Where available, species-specific TEFs for each tissue were used in order to obtain diet-corrected stable isotope ratios (electronic supplementary material, table S3a). In most cases, however, species-specific Δ_tissue-diet_ were unavailable, and so average TEFs were calculated for each tissue using the literature data for related taxa with similar diets (electronic supplementary material, table S3b). The individual isotopic compositions for each sample prior to applying TEF values are listed in electronic supplementary material, tables S4 and S5. Bioapatite *δ*^18^O data represent either bioapatite structural carbonate oxygen or laser-produced total-oxygen (LPTO), depending on the particular sample/method. LPTO data represent a combination of phosphate + carbonate + hydroxyl oxygen, though the hydroxyl contribution is minimal per [73], leading to LPTO being a weighted average of these sources, and typically approximately 1‰ higher than the phosphate *δ*^18^O alone (i.e. by 1.2‰ per Cerling & Sharp [112] and by 0.7‰ per Sharp & Cerling [118]). Comparisons to previously reported relationships [73,112,118] that are consistent with the analysed dataset (electronic supplementary material, figure S1*a*) allow bioapatite oxygen isotope data from structural carbonate and LPTO to be converted to phosphate oxygen isotope equivalents to facilitate comparison and discussion.

### 2.7. Data analyses

Data analyses were performed, and base plots made, using the R programming language and contained core packages [119], along with the functions in the ‘ggplot2’ package [120], with plots further modified or grouped using Adobe Illustrator CS6. Statistical tests were performed to assess normality and distribution of the data, and to confirm the comparability of isotopic data from different tissues and individuals in the species-level community comparisons (electronic supplementary material, S1, figures S1 & S2 and tables S6 & S7).

Community-level comparisons were performed on the stable carbon, nitrogen and oxygen isotope results for all sampled species. Stable isotope data were adjusted through the application of TEFs (for nitrogen and carbon) and carbonate-phosphate/LPTO-phosphate discrimination factors (for oxygen), as described above (and in electronic supplementary material, S1). Comparisons were made using combined keratin (hair and claw) and bioapatite (bone and tooth) data, respectively. In each case, species isotopic ranges were plotted for keratin nitrogen, keratin carbon, bioapatite carbon and bioapatite oxygen to test for species-level differences, and illustrated in combined bi-plots of isotopic ratios (keratin nitrogen versus keratin carbon, bioapatite carbon versus bioapatite oxygen) to more fully visualize the range of community isotopic distribution and possibly niche space occupation, under the methodological constraints present in a Mesozoic system (i.e. no preservation of blood/muscle tissue, little to no preservation of measurable plant or soft-bodied organism tissues, general lack of measurable/observable diet data). These distributions were also compared with observational natural history records compiled from the literature (electronic supplementary material, table S2). These comparisons were used to assess the inferences made from the distributions themselves (nitrogen isotope results being used in somewhat the same way relative to carbon isotope or oxygen isotope results), given the general lack of availability of such data in fossil systems.

Body water oxygen isotope concentrations were calculated from bioapatite oxygen isotope data (adjusted to their phosphate equivalent) for each sampled taxon, using a combination of taxon/physiology-specific equations from Kohn [121] (for endotherms; faunivorous/carnivorous mammals, omnivorous mammals and herbivorous mammals) and Amiot *et al*. [65] (for ectotherms; reptiles, fish):

— faunivorous mammal (*δ*^18^O_surface water_ = (*δ*^18^O_phosphate_ − 21.3 + 3*h*)/0.74),
— omnivorous mammal (*δ*^18^O_surface water_ = (*δ*^18^O_phosphate_ − 22.7 + 3.9*h*)/0.64),
— herbivorous mammal (*δ*^18^O_surface water_ = (*δ*^18^O_phosphate_ − 26.8 + 8.9*h*)/0.76),
— reptile/fish (*δ*^18^O_surface water_ = 0.82*δ*^18^O_phosphate_ − 19.13),

where *δ*^18^O_phosphate_ is the bioapatite oxygen from the sampled taxon, and *h* is the decimal fraction relative humidity (in this case obtained from the average annual relative humidity for the sampling area, approx. 75%).

These results were then converted into temperature using the following equation in Kohn [121]:

$$\delta^{18}O_{\mathrm{surface}\;\mathrm{water}}=(0.69)(T)-13.6,$$

where *T* is the temperature in degrees Celsius.

In addition, we propose a modification to the two-part temperature calculation using combined oxygen isotope data from endotherms and ectotherms from Fricke & Wing [25], in order to calculate a mean temperature estimate using all sampled taxa. The original equations are

$$\begin{array}{rl} & \delta^{18}O_{\mathrm{mammal}}=(0.76)(\delta^{18}O_{\mathrm{river}})+19.94\\ & T=111.4-(4.3)(\delta^{18}O_{\mathrm{gar}\;\mathrm{phosphate}}-\delta^{18}O_{\mathrm{river}}). \end{array}$$

The first part uses the Kohn [121] mammalian herbivore equation (assuming *h* = approx. 0.75), and then combines the resultant river oxygen isotope composition with the oxygen isotope data for ectothermic fish (in their case derived from fossil gar ganoine) in order to calculate river temperature. This procedure was modified to calculate *δ*^18^O_river_ for each individual endothermic species using the equations noted above, with the mean of the resultant *δ*^18^O_river_ values entered into the temperature equation, and with the mean *δ*^18^O_phosphate_ of all ectothermic taxa used in place of using a single gar taxon. Mammalian physiology/body temperature is considered in these calculations via the Kohn endotherm equations (though variability due to slight differences in physiology and humidity tolerance in the averaged mammal samples is probably not fully accounted for due to the pooled nature of the calculation). As noted above, individual taxon temperature estimates were also calculated using species-specific or diet/physiology-specific equations where available, allowing for comparison with the results of the modified two-part endotherm–ectotherm mean temperature calculation. In all cases, calculated temperatures were also compared with water temperatures recorded hourly for the Atchafalaya River, obtained from the United States Geological Survey (site 07381600 Lower Atchafalaya River at Morgan City, LA) [122], covering the dates 24 February 2015–22 February 2018, inclusive (electronic supplementary material, table S8).

## 3. Results

### 3.1. Individual and intraspecific variation

See electronic supplementary material, S1 for detailed descriptions of the analytical results of all tissue comparisons and methodological testing. Isotopic results for nitrogen, carbon and oxygen all fall within broadly expected ranges based on related mammals, reptiles and fish, though the degree of analytical variability was dependent on several factors. In general, variability was low in repeated sampling of the same tissues of a given specimen, but became higher when comparing between tissues, or between specimens and species. Bulk powder isotopic analyses produced more consistent, low variability, results, whereas laser ablation isotopic analyses resulted in higher variation (electronic supplementary material, table S5). This is probably due to the nature of the sampling and analytical methods, as bulk powders naturally lead to a more consistently mixed and averaged isotopic signal, whereas laser ablation, by nature of the small spatial scale of the samples, potentially reveals temporal variability in isotopic signal. This higher variability is consistent with previous stable isotope studies using laser ablation techniques [73,123].

When comparing carbon isotopic compositions taxonomically, variation was greater in certain species when compared with others in the overall sample. Within the keratin samples, *Sus* (feral pig) and *Mephitis* (skunk) exhibited the greatest variation in carbon isotope compositions. Within the bioapatite samples, the greatest degree of isotopic variation was seen in aquatic taxa, particularly *Atractosteus* (alligator gar) and *Lepisosteus* (longnose/spotted gar). Offsets between keratin and bioapatite isotopic compositions in mammalian taxa match literature expectations (electronic supplementary material, table S3). While no direct *Alligator* hard tissue to soft tissue comparison could be found in the literature, the keratin-bioapatite offsets in sampled *Alligator* match those of other known reptiles (electronic supplementary material, tables S3 and S5). While keratin and bioapatite carbon isotopic ranges were generally consistent in taxa sampled for both, there were moderate differences in average signal recorded in several taxa, including *Neovison* (mink), *Lontra* (otter) and *Sciurus* (squirrel).

Offsets in oxygen isotopic compositions of bioapatite tissues obtained via bulk powder carbonate sampling and laser ablation total oxygen sampling for the same specimens were on average approximately 7‰, consistent with the literature expectations [73,112,118,123] (also see electronic supplementary material, S1). However, oxygen isotope compositions obtained from bone carbonate and tooth enamel, both sampled by laser ablation, contained the highest levels of variation seen in the study. This issue is discussed in greater detail in electronic supplementary material, S1, but it is likely that this higher variability represents differences in the temporal scale reflected in these tissues, and so more acutely corresponds to seasonal and ontogenetic changes experienced in the lives of the sampled organisms.

### 3.2. Community comparisons

**Figure 2.**
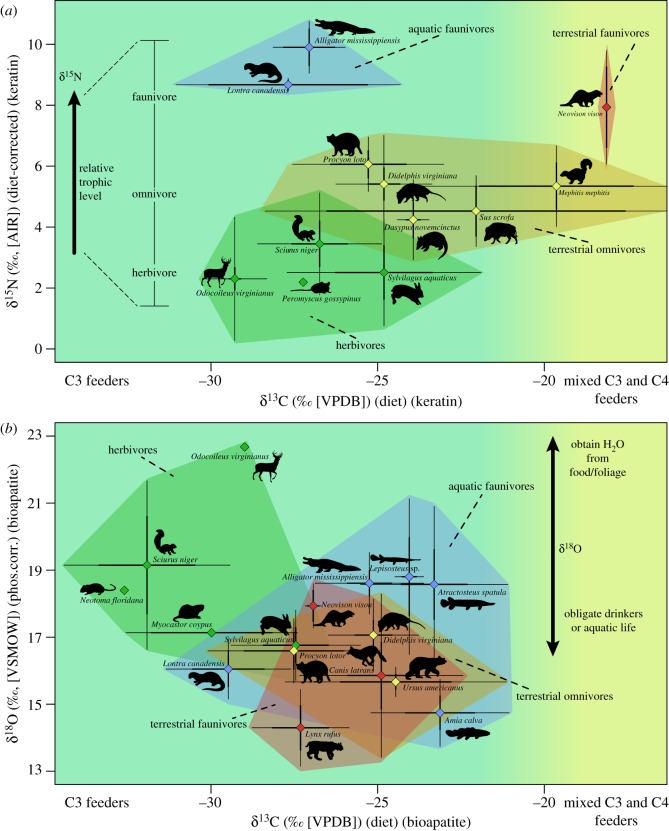
Bi-plots of (*a*) keratin *δ*^15^N ([AIR], diet) versus keratin *δ*^13^C ([VPDB], diet) and (*b*) bioapatite *δ*^18^O ([VSMOW], phosphate-corrected) versus bioapatite *δ*^13^C ([VPDB], diet) community distributions for all sampled taxa. Species means plotted with standard errors (thick lines) and standard deviations (thin lines). Coloured hulls indicate diet/guild assignments (green = herbivore, yellow = omnivore, red = terrestrial faunivore, blue = aquatic faunivore). See table 2 for mean isotopic compositions for keratin, table 3 for mean isotopic compositions for bioapatite, and electronic supplementary material, table S2 for natural history data for each taxon.

Mean isotopic results from bioapatite and keratin samples of each specimen, with TEFs applied, are presented in table 1. TEF-corrected mean isotopic results for each taxon (+/− standard error) are presented for keratin in table 2 and for bioapatite in table 3. Bi-plots of keratin *δ*^15^N and *δ*^13^C (figure 2*a*), and bioapatite *δ*^13^C and *δ*^18^O (figure 2*b*) were produced to assess the range and variation in isotopic compositions for each taxon, and visualize resource/niche partitioning in this community in multi-dimensional space. Coloured hulls in these figures (with associated colour-filled points) indicate broad dietary/guild assignment (green = herbivore, yellow = omnivore, red = terrestrial faunivore, blue = aquatic faunivore). Keratin *δ*^15^N effectively separated groups of taxa by relative trophic position (figure 2*a*) over a range of approximately 0 to + 10‰, with faunivorous taxa (e.g. *Alligator, Lontra, Neovison*) possessing mean *δ*^15^N > + 7.5‰, omnivorous taxa (e.g. *Procyon, Didelphis, Mephitis, Dasypus, Sus*) possessing mean *δ*^15^N of +4‰ to +6.5‰, and herbivorous taxa (e.g. *Peromyscus, Sciurus, Sylvilagus, Odocoileus*) possessing mean *δ*^15^N < +4‰. In both keratin *δ*^13^C (figure 2*a*) and bioapatite *δ*^13^C (figure 2*b*), taxa occupied similar ranges of isotopic space (approx. –30 to –15‰ for keratin *δ*^13^C and –35 to –20‰ for bioapatite *δ*^13^C). No significant outlier taxa are present in the bioapatite *δ*^13^C results, whereas three taxa in the keratin dataset (*Neovison*, *Mephitis* and *Sus*, with the latter two also having much higher variability than observed in other taxa) had average *δ*^13^C that were approximately 4 to 8‰ higher than the mean of most sampled taxa. Herbivores typically had lower *δ*^13^C than most other taxa, though moderate to considerable overlap exists in *δ*^13^C among most taxa for both keratin and bioapatite. Fully aquatic faunivores (i.e. fish) had higher *δ*^13^C than most other faunivores, and little difference existed in *δ*^13^C distributions for sampled terrestrial faunivores and omnivores. A high degree of overlap also existed in bioapatite *δ*^18^O, with a total range of approximately +13 to +23‰. Within this range, omnivores and faunivores typically had the lowest *δ*^18^O, followed by fish and herbivores. The main exceptions to this pattern were the high values for a large herbivore, *Odocoileus* (mean *δ*^18^O of approx. +23‰), and low values of *Amia* (mean *δ*^18^O of approx. +15‰) when compared with other fish or semi-aquatic taxa.

**Table 1.** Specimen-level mean stable isotope compositions, subdivided by tissue sampled. *δ*^13^C values standardized to VPDB and TEF-corrected to diet. *δ*^15^N values standardized to AIR and TEF-corrected to diet. *δ*^18^O values standardized to VSMOW and corrected to phosphate moiety.

specimen no.	taxon	*δ*^13^C (‰ [VPDB]) (diet) (bioapatite)	*δ*^13^C (‰ [VPDB]) (diet) (keratin)	*δ*^18^O (‰, [VSMOW]) (phos.) (bioapatite)	*δ*^15^N (‰, [AIR]) (diet) (keratin)
AWR-105	*Alligator mississippiensis*	−25.4	−26.3	+19.1	+10.5
AWR-107	*Alligator mississippiensis*	−27.1	−27.8	+18.0	+9.3
AWR-128	*Alligator mississippiensis*	−23.8	—	+17.6	—
AWR-134	*Alligator mississippiensis*	−22.8	—	+18.8	—
AWR-135	*Alligator mississippiensis*	−25.9	—	+18.1	—
AWR-96	*Alligator mississippiensis*	−26.4	—	+20.1	—
AWR-123	*Amia calva*	−20.2	—	+15.2	—
AWR-124	*Amia calva*	−23.1	—	+13.8	—
AWR-125	*Amia calva*	−24.8	—	+14.0	—
AWR-126	*Amia calva*	−24.4	—	+15.9	—
AWR-106	*Atractosteus spatula*	−20.4	—	+20.6	—
AWR-120	*Atractosteus spatula*	−23.4	—	+21.6	—
AWR-127	*Atractosteus spatula*	−26.6	—	+17.0	—
AWR-136	*Atractosteus spatula*	−23.5	—	+16.9	—
AWR-137	*Atractosteus spatula*	−22.7	—	+16.9	—
AWR-129	*Canis latrans*	−24.8	—	+19.5	—
AWR-156	*Canis latrans*	−27.2	—	+18.3	—
LSUMZ-11274	*Canis latrans*	−22.5	—	+13.6	—
LSUMZ-17873	*Canis latrans*	−21.5	—	+14.8	—
LSUMZ-26849	*Canis latrans*	−25.9	—	+13.8	—
LSUMZ-26851	*Canis latrans*	−27.4	—	+15.1	—
AWR-05	*Dasypus novemcinctus*	—	−23.8	—	+2.3
AWR-18	*Dasypus novemcinctus*	—	−23.3	—	+4.1
AWR-26	*Dasypus novemcinctus*	—	−23.8	—	+5.9
AWR-28	*Dasypus novemcinctus*	—	−24.0	—	+4.5
AWR-29	*Dasypus novemcinctus*	—	−24.7	—	+4.4
AWR-02	*Didelphis virginiana*	−25.9	−25.7	+17.5	+4.3
AWR-06	*Didelphis virginiana*	−25.0	−23.1	+16.9	+5.8
AWR-07	*Didelphis virginiana*	−27.4	−26.8	+16.1	+8.5
AWR-08	*Didelphis virginiana*	−23.8	−25.9	+15.8	+6.3
AWR-119	*Didelphis virginiana*	−24.5	−24.0	+18.7	+3.1
AWR-12	*Didelphis virginiana*	−24.0	−24.8	+17.5	+4.2
AWR-13	*Didelphis virginiana*	—	−24.3	—	+5.5
AWR-22	*Didelphis virginiana*	—	−26.0	—	+4.4
AWR-27	*Didelphis virginiana*	—	−22.6	—	+6.6
AWR-121	*Lepisosteus* sp.	−24.2	—	+15.4	—
AWR-122	*Lepisosteus* sp.	−23.7	—	+19.4	—
AWR-35	*Lepisosteus* sp.	−24.6	—	+20.2	—
AWR-37	*Lepisosteus* sp.	−23.7	—	+20.3	—
AWR-09	*Lontra canadensis*	−30.8	−30.1	+16.6	+8.5
AWR-130	*Lontra canadensis*	−30.3	—	+16.5	—
AWR-45	*Lontra canadensis*	−27.3	−25.3	+15.0	+8.8
LSUMZ-13747	*Lynx rufus*	−27.0	—	+13.9	—
LSUMZ-32761	*Lynx rufus*	−28.9	—	+15.6	—
LSUMZ-9726	*Lynx rufus*	−26.0	—	+13.4	—
AWR-20	*Mephitis mephitis*	—	−16.2	—	+5.3
AWR-32	*Mephitis mephitis*	—	−18.7	—	+4.0
AWR-33	*Mephitis mephitis*	—	−24.0	—	+6.7
AWR-102	*Myocastor coypus*	−28.2	—	+17.1	—
AWR-99	*Myocastor coypus*	−31.8	—	+17.2	—
AWR-133	*Neotoma floridana*	−32.6	—	+18.4	—
AWR-19	*Neovison vison*	−26.8	−18.1	+17.5	+6.6
AWR-46	*Neovison vison*	−27.1	−18.1	+18.4	+9.3
AWR-17	*Odocoileus virginianus*	—	−30.4	—	+2.6
AWR-21	*Odocoileus virginianus*	−29.0	−28.8	+22.7	+1.3
AWR-50	*Odocoileus virginianus*	—	−29.1	—	+3.0
AWR-51	*Odocoileus virginianus*	—	−30.5	—	+3.5
AWR-52	*Odocoileus virginianus*	—	−29.6	—	+2.6
AWR-55	*Odocoileus virginianus*	—	−30.0	—	+0.3
AWR-56	*Odocoileus virginianus*	—	−28.8	—	+1.2
AWR-57	*Odocoileus virginianus*	—	−28.4	—	+2.4
AWR-58	*Odocoileus virginianus*	—	−28.2	—	+7.2
AWR-59	*Odocoileus virginianus*	—	−29.8	—	+8.2
AWR-60	*Odocoileus virginianus*	—	−30.1	—	+0.8
AWR-61	*Odocoileus virginianus*	—	−31.2	—	+3.8
AWR-62	*Odocoileus virginianus*	—	−31.1	—	+4.4
AWR-63	*Odocoileus virginianus*	—	−29.9	—	+1.6
AWR-64	*Odocoileus virginianus*	—	−30.6	—	+5.9
AWR-65	*Odocoileus virginianus*	—	−28.8	—	+2.8
AWR-66	*Odocoileus virginianus*	—	−29.9	—	+2.8
AWR-67	*Odocoileus virginianus*	—	−28.7	—	+0.4
AWR-68	*Odocoileus virginianus*	—	−27.9	—	+1.8
AWR-69	*Odocoileus virginianus*	—	−29.5	—	−0.4
AWR-70	*Odocoileus virginianus*	—	−29.2	—	+2.1
AWR-71	*Odocoileus virginianus*	—	−29.7	—	+1.8
AWR-72	*Odocoileus virginianus*	—	−28.9	—	+2.4
AWR-73	*Odocoileus virginianus*	—	−28.4	—	+2.2
AWR-74	*Odocoileus virginianus*	—	−28.7	—	+1.2
AWR-75	*Odocoileus virginianus*	—	−28.5	—	+1.0
AWR-76	*Odocoileus virginianus*	—	−28.8	—	−0.2
AWR-77	*Odocoileus virginianus*	—	−28.8	—	+1.7
AWR-78	*Odocoileus virginianus*	—	−27.0	—	+1.1
AWR-79	*Odocoileus virginianus*	—	−29.2	—	−0.3
AWR-24	*Peromyscus gossypinus*	—	−27.2	—	+2.2
AWR-01	*Procyon lotor*	−27.0	−25.5	+17.4	+5.7
AWR-10	*Procyon lotor*	−25.2	−22.1	+15.5	+6.0
AWR-25	*Procyon lotor*	—	−27.3	—	+5.2
AWR-30	*Procyon lotor*	—	−26.3	—	+7.3
AWR-98	*Procyon lotor*	−30.3	—	+16.8	—
AWR-104	*Sciurus niger*	−34.5	—	+19.3	—
AWR-11	*Sciurus niger*	−31.8	−26.1	+16.5	+6.0
AWR-23	*Sciurus niger*	—	−27.9	—	+1.6
AWR-31	*Sciurus niger*	−29.5	−27.3	+21.6	+2.6
AWR-34	*Sciurus niger*	—	−23.8	—	+4.0
AWR-36	*Sciurus niger*	—	−28.6	—	+2.9
AWR-48	*Sus scrofa*	—	−17.6	—	+5.3
AWR-85	*Sus scrofa*	—	−26.5	—	+3.7
AWR-101	*Sylvilagus aquaticus*	−27.0	−24.4	+15.4	+3.0
AWR-47	*Sylvilagus aquaticus*	−27.9	−28.2	+16.7	+0.7
AWR-86	*Sylvilagus aquaticus*	−25.1	−21.2	+18.0	+4.7
AWR-93	*Sylvilagus aquaticus*	−29.8	−25.4	+16.9	+1.5
LSUMZ-36097	*Ursus americanus*	−26.9	—	+16.0	—
LSUMZ-36101	*Ursus americanus*	−22.5	—	+15.6	—
LSUMZ-36102	*Ursus americanus*	−20.9	—	+16.0	—
LSUMZ-9045	*Ursus americanus*	−27.7	—	+15.1	—

**Table 2.** Species-level mean *δ*^13^C_diet_ and *δ*^15^N_diet_ values from keratin samples. *δ*^13^C values standardized to VPDB and TEF-corrected to diet. *δ*^15^N values standardized to AIR and TEF-corrected to diet.

taxon	mean *δ*^13^C (‰, VPDB) (diet)	std. err. *δ*^13^C (‰)	mean *δ*^15^N (‰, AIR) (diet)	std. err. *δ*^15^N (‰)
*Alligator mississippiensis*	−27.1	0.77	+9.9	0.60
*Lontra canadensis*	−27.7	2.38	+8.7	0.14
*Neovison vison*	−18.1	0.01	+7.9	1.32
*Procyon lotor*	−25.3	1.13	+6.1	0.45
*Didelphis virginiana*	−24.8	0.48	+5.4	0.53
*Mephitis mephitis*	−19.6	2.31	+5.3	0.76
*Dasypus novemcinctus*	−23.9	0.21	+4.2	0.59
*Sus scrofa*	−22.0	4.48	+4.5	0.82
*Peromyscus gossypinus*	−27.2	—	+2.2	—
*Sciurus niger*	−26.7	0.83	+3.4	0.75
*Sylvilagus aquaticus*	−24.8	1.45	+2.5	0.88
*Odocoileus virginianus*	−29.3	0.18	+2.3	0.37

**Table 3.** Species-level mean *δ*^13^C_diet_ and *δ*^18^O_phosphate_ for bioapatite samples. *δ*^13^C values standardized to VPDB and TEF-corrected to diet. *δ*^18^O values standardized to VSMOW and corrected to phosphate moiety.

taxon	mean *δ*^13^C (‰, VPDB) (diet)	std. err. *δ*^13^C (‰)	mean *δ*^18^O (‰, VSMOW)(phos.)	std. err. *δ*^18^O (‰)
*Amia calva*	−23.1	1.04	+14.7	0.50
*Atractosteus spatula*	−23.3	0.99	+18.6	1.04
*Lepisosteus* sp.	−24.1	0.22	+18.8	1.16
*Alligator mississippiensis*	−25.3	0.66	+18.6	0.37
*Lontra canadensis*	−29.5	1.08	+16.0	0.52
*Neovison vison*	−26.9	0.17	+17.9	0.47
*Lynx rufus*	−27.3	0.84	+14.3	0.66
*Canis latrans*	−24.9	1.00	+15.9	0.99
*Ursus americanus*	−24.5	1.66	+15.7	0.22
*Didelphis virginiana*	−25.1	0.55	+17.1	0.44
*Procyon lotor*	−27.5	1.49	+16.6	0.55
*Neotoma floridana*	−32.6	—	+18.4	—
*Sciurus niger*	−31.9	1.44	+19.1	1.45
*Myocastor coypus*	−29.9	1.82	+17.1	0.03
*Sylvilagus aquaticus*	−27.5	0.97	+16.8	0.52
*Odocoileus virginianus*	−28.9	—	+22.7	—

### 3.3. Temperature reconstructions

**Figure 3.**
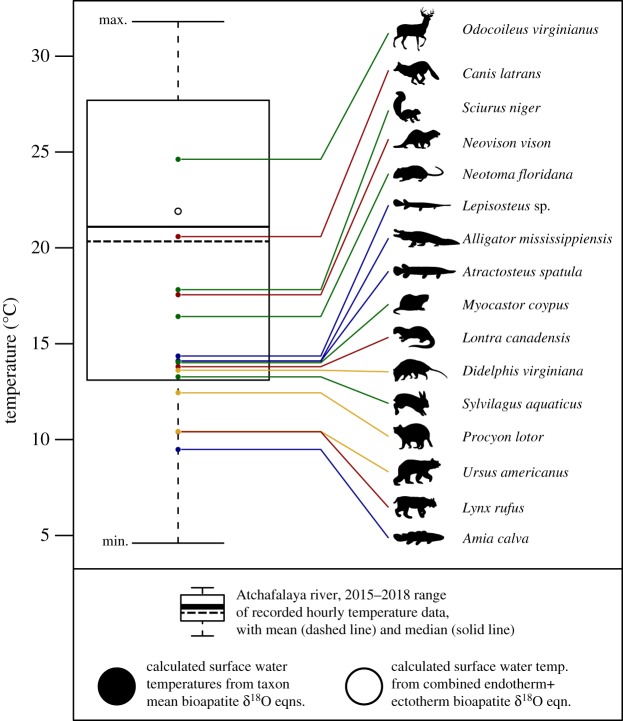
Comparison of Atchafalaya River measured annual water temperature range (median = solid line, mean = dashed line) with calculated water temperature (filled circles) from mean bioapatite oxygen isotope data for each measured taxon (using taxon/physiology specific equations from Kohn [121] and Amiot *et al.* [65]). Also plotted (unfilled circle) is the temperature estimate using modified two-point calculation of Fricke & Wing [25] derived from mean bioapatite oxygen isotope data of endotherms (mammals) and ectotherms (fish + reptiles). See table 4 for calculated and measured temperature values.

Water temperatures for each taxon were calculated using equations from Kohn [121] and Amiot *et al.* [65], and compared against water temperatures recorded hourly between 2015 and 2018 from the Atchafalaya River near Morgan City, LA (table 4 and figure 3; electronic supplementary material, table S8). Mean annual Atchafalaya River water temperature was 20.3°C, with temperatures ranging from a minimum winter water temperature of 4.6°C to a maximum summer water temperature of 35.5°C. Temperatures calculated from mean bioapatite oxygen isotope data varied among taxa, though all fell within the annual water temperature range for the Atchafalaya River. Temperatures calculated from oxygen isotope results for *Canis latrans* most closely reflected mean annual river temperature (20.6 versus 20.3°C), and the majority of bioapatite-derived temperatures plotted between 10 and 15°C. This is particularly true of temperatures derived from aquatic ectotherm bioapatite, in which all sampled taxa produced estimates consistent with non-summer Atchafalaya River water temperature ranges. When the proposed multi-taxic two-part endotherm–ectotherm method (modified from Fricke & Wing [25]) is used to calculate mean water temperature obtained from the bioapatite oxygen isotope results of all taxa, a value of 21.9°C is obtained. This value is within 2°C of the measured mean annual temperature (20.3°C) and 1°C of the measured median annual temperature (21.1°C).

**Table 4.** Calculated temperature and *δ*^18^O_water_ for bioapatite from each sampled taxon, along with measured Atchafalaya River water temperatures derived from USGS data (electronic supplementary material, table S8; [122]). Bioapatite *δ*^18^O values standardized to VSMOW and corrected to phosphate moiety. Method of calculation or source of measured data noted in ‘details’ column, with referenced papers discussed in manuscript text.

datapoint(s)	temp. (°C)	*δ*^18^O_w_ (‰)	details
Atch. Riv. Max. Rec. Temp.	31.8	—	USGS site 07381600 Lower Atchafalaya River at Morgan City, LA (2015–2018) [122]
*Odocoileus virginianus*	24.6	3.4	Kohn 1996 mammalian herbivore eqn. [121]
All Taxa Mean Endo-Ecto Calc.	21.9	—	Modified Fricke & Wing 2004 two-part endotherm–ectotherm water temp calculation [121]
Atch. Riv. Median Rec. Temp.	21.1	—	USGS site 07381600 Lower Atchafalaya River at Morgan City, LA (2015–2018) [122]
*Canis latrans*	20.6	0.6	Kohn 1996 mammalian carnivore eqn. [121]
Atch. Riv. Mean Rec. Temp.	20.3	—	USGS site 07381600 Lower Atchafalaya River at Morgan City, LA (2015–2018) [122]
*Sciurus niger*	17.8	−1.3	Kohn 1996 mammalian herbivore eqn. [121]
*Neovison vison*	17.6	−1.5	Kohn 1996 mammalian carnivore eqn. [121]
*Neotoma floridana*	16.4	−2.3	Kohn 1996 mammalian herbivore eqn. [121]
*Lepisosteus* sp.	14.4	−3.7	Amiot 2007 ectotherm eqn. [65]
*Alligator mississippiensis*	14.1	−3.9	Amiot 2007 ectotherm eqn. [65]
*Atractosteus spatula*	14.1	−3.9	Amiot 2007 ectotherm eqn. [65]
*Myocastor coypus*	14.0	−3.9	Kohn 1996 mammalian herbivore eqn. [121]
*Lontra canadensis*	13.8	−4.1	Kohn 1996 mammalian carnivore eqn. [121]
*Didelphis virginiana*	13.6	−4.2	Kohn 1996 mammalian omnivore eqn. [121]
*Sylvilagus aquaticus*	13.3	−4.4	Kohn 1996 mammalian herbivore eqn. [121]
*Procyon lotor*	12.4	−5.0	Kohn 1996 mammalian omnivore eqn. [121]
*Ursus americanus*	10.4	−6.4	Kohn 1996 mammalian omnivore eqn. [121]
*Lynx rufus*	10.4	−6.4	Kohn 1996 mammalian carnivore eqn. [121]
*Amia calva*	9.5	−7.1	Amiot 2007 ectotherm eqn. [65]
Atch. Riv. Min. Rec. Temp.	4.6	—	USGS site 07381600 Lower Atchafalaya River at Morgan City, LA (2015–2018) [122]

## 4. Discussion

### 4.1. Factors affecting isotopic compositions of vertebrate tissue

The relative amounts of different stable isotopes preserved in the tissues of an organism depend on several factors. Stable carbon isotope ratios, particularly in terrestrial systems, generally relate to the plant source at the base of the food web, which typically follow either the C_3_ (*δ*^13^C∼–36 to –21‰, average of approx. –28 to –26‰), or C_4_ (*δ*^13^C∼–14 to –10‰, average∼–12‰) photosynthetic pathway, being therefore low in *δ*^13^C relative to modern atmospheric CO_2_ (approx. –8.5 to –8.4‰) [1,4,10,20,29,39,60,78,111,124]. The stable carbon isotope compositions of ingested plant tissues do not generally change greatly per trophic level when they are incorporated into the tissues of herbivores and, later, omnivores/generalists and faunivores (i.e. organisms regularly/primarily feeding on invertebrates and vertebrates) [2,8,10,125]. Stable carbon isotope ratios are also affected by body size [126], physiology [5] and local environmental conditions, including humidity [5,20], forest density/canopy cover [55,127], dissolved inorganic carbon (DIC) in surrounding water bodies [111], and degree of marine/coastal influence [10,111]. Nitrogen isotope ratios reflect the relative trophic level of a sampled organism in the local food web, with ^15^N becoming more enriched as the trophic level increases [1,8,9,111]. This predictable enrichment (most often approx. 2–4‰) per trophic level makes nitrogen isotope ratios a powerful tool for studying diet, food web interactions and resource partitioning [1,3,16,48,50–54,56,82,90,92,103,106,128–130]. Depending on the study system, the utilization of nitrogen isotopes in (palaeo)ecological studies may be limited, given that it is not contained in the inorganic components of bone and teeth [3,4,8,49,78,111,131]. Oxygen isotope ratios are perhaps the most complex of those included in this study, being controlled by a number of factors, most largely related to environmental and physiological conditions [4,5,20,49,111]. Environmentally, changes in *δ*^18^O record consumed water sources, depending on the diet/behaviour of an organism, and are often closely related to surface water conditions, local and regional patterns of temperature, precipitation, humidity, evaporation, elevation and marine versus freshwater settings [5,6,20,121]. The behaviour and physiology of a given organism influences which of these external environmental changes to *δ*^18^O may predominate in the signal present in a given tissue. These considerations include whether an organism obtains water by drinking (recording a value closer to the surface water *δ*^18^O) or primarily through its diet (such as large herbivores obtaining water through consuming plant matter rather than drinking, in which the more ^18^O-rich plant water from leaves will be reflected in the tissues of the herbivore), the degree of humidity dependence in a given taxon, and metabolism (i.e. ectothermic versus endothermic) of a taxon [4,5,20,49]. These various factors must be considered when interpreting the isotopic results in the sampled taxa, and the conclusions drawn from them regarding the community ecology of the Atchafalaya system. The major observations are discussed below, with additional discussion of tissue-level isotopic results found in electronic supplementary material, S1.

### 4.2. Isotope ecology of Atchafalaya taxa

Comparisons of mean isotope distributions (figure 2) illustrate the considerable variability and overlap in isotopic resource usage among sampled Atchafalaya taxa. While non-overlap of species isotopic ranges is not required, or even necessarily expected, a considerable degree of overlap in isotopic range without significant differences in mean isotope compositions suggests that dietary or trophic discriminations may not be resolvable using these isotopic proxies alone. Where additional data are present (such as natural history or other observational records), then these distributions may be further contextualized and meaningfully interpreted. As will be discussed further below, this has potential implications for studies of Mesozoic systems, where additional sources of contextual information may or may not be present.

The degree of overlap present, and ecological interpretation possible, in these data is dependent on the particular isotope and tissue being discussed. Keratin *δ*^15^N is more effective at partitioning taxa by relative trophic level (low values representing primary consumers and higher values representing secondary and tertiary consumers), matching predictions from natural history data (electronic supplementary material, table S2). Both keratin and bioapatite *δ*^13^C have considerable overlap in terms of overall distribution, among guilds, as well as separately among the sampled species (figure 2), and their strongly negative values probably reflect C_3_-based systems [1,4,111]. A minor exception to this exists in the keratin *δ*^13^C ranges of *Neovison* (mink), *Mephitis* (skunk) and *Sus* (feral pig), all of which are richer in ^13^C relative to the community average, suggesting some degree of C_4_ influence on their diets. Interestingly, the mean bioapatite *δ*^13^C of *Neovison* is approximately –27‰, whereas its keratin *δ*^13^C is approximately –18‰, suggesting that whatever C_4_ influence existed on the diet of *Neovison* in this sample may have been present on a shorter/recent timescale (keratin isotopic signals typically reflect weeks to months [77,132]) when compared with the more permanent or longer-term *δ*^13^C signal provided from bioapatite. Mammalian enamel bioapatite is generally fixed relatively early in life, often over a period of several months to half a year [133], whereas bone bioapatite reflects something akin to a multi-year rolling average, taking a decade in some cases to fully remodel [134]). It is also possible that *Neovison* (mink), which is known to feed on a wide variety of terrestrial and aquatic organisms, shifts its diet opportunistically or seasonally between C_3_ (e.g. fish, muskrat, swamp rabbit etc.) and C_4_ (e.g. animals feeding on grass or human grain crops, such as some rodents, birds or insects) feeding prey, and the C_3_ source happens to have been what was preserved in these individuals during the relatively short period of mammalian enamel formation early in ontogeny [133].

Despite the observed overlaps, some broad patterns can be seen in the keratin and bioapatite carbon isotope results at both the species and guild levels. Among larger grouping, such as diet/guild (coloured hulls in figure 2), some separation can be seen between herbivores and faunivores + omnivores, as well as between fully aquatic faunivores (i.e. fish) and other faunivores + omnivores. These relative differences are more pronounced in the bioapatite carbon isotope results than in the keratin carbon isotope compositions, which as noted above may relate to temporal effects or dietary averaging in bioapatite versus keratin isotope signatures.

Relatively little evidence exists to suggest a widespread canopy effect contribution to the overall pattern of either the keratin or bioapatite *δ*^13^C [55,127]. It is possible, however, that canopy effects could explain the lower mean *δ*^13^C of *Sciurus niger* and *Neotoma floridana*, in particular, relative to both other herbivores and the majority of taxa sampled in this study, as both are known to be either arboreal or prefer forest understorey habitats [135,136]. Another potential cause of the distinct *δ*^13^C of these taxa when compared with the other sampled herbivores is their more granivorous diet, whereas *Odocoileus virginianus* is primarily a terrestrial folivore, and *Sylvilagus aquaticus* and *Myocastor coypus* consume a mixture of terrestrial and aquatic vegetation (see electronic supplementary material, table S2). Previous studies comparing differences in the carbon isotope compositions of photosynthetic versus reproductive plant tissues, however, typically find the latter (seeds, fruit etc.) to be ^13^C-enriched rather than ^13^C-depleted [137], suggesting that dietary effects may be at odds with potential canopy effects. A canopy effect explanation for *Sciurus* and *Neotoma* is also supported by the relatively higher mean bioapatite *δ*^18^O for these taxa, as arboreality has previously been shown to correlate with ^18^O-enrichment [49].

Among higher-level consumers, several species-level patterns exist. Omnivorous species such as *Procyon* and *Didelphis* contain overlapping isotopic ranges, though the former overlaps with multiple herbivorous taxa, whereas the latter does not (overlapping only with *Sylvilagus*). This difference may be the result of aquatic invertebrates/bivalves forming a large component of the diet of *Procyon* when compared with *Didelphis* (electronic supplementary material, table S2), as similar diets have previously been found to be more negative in *δ*^13^C [138]. Relatively close isotopic associations exist between some predators and potential prey *δ*^13^C (e.g. *Lynx* and *Sylvilagus*, *Canis* and *Didelphis*) [139], though there are also cases where predator–prey relationships are very difficult to extrapolate based on the available data (e.g. *Alligator*, *Ursus*, *Lontra*). In the case of both *Alligator* and *Ursus* (bear), it is known that these animals can have varied diets composed of terrestrial and/or aquatic sources [140,141], and in the case of *Ursus* there is also probably a large herbivorous component to the diet [140]. The *δ*^13^C range of *Ursus* structural carbonate from bone bioapatite in this study is interesting as it contrasts with recent reports [140] of their dietary preferences from other areas in Louisiana, where C_4_ plants (specifically corn) seem to make up a large component of the overall diet. It is possible that these *Ursus* specimens did not feed as heavily on human crops. Given the seasonal nature of that feeding [140], it is also possible that the averaging effect of the sampled bone bioapatite removes any apparent C_4_ effects when combined with primarily C_3_ feeding during the majority of the year. Crustaceans, fish (e.g. *Amia*, *Atractosteus*) and mammals (e.g. *Myocastor*) are typical prey for *Alligator* in Louisiana [141], forming a large component of their diet. This is consistent with the intermediate *δ*^13^C range of *Alligator* relative to *Myocastor* and sampled fish taxa. The three sampled fish taxa (*Amia*, *Atractosteus* and *Lepisosteus*) have carbon isotope ranges consistent with aquatic feeding on pelagic/limnetic and littoral, rather than benthic, food sources [110,138,142,143]. The measured carbon isotope ranges of *Lontra*, being depleted of ^13^C relative to other aquatic taxa and highly variable (for keratin), may be the result of several factors. Primarily freshwater feeding in *Lontra* would explain the lower *δ*^13^C if individuals of other aquatic taxa (e.g. *Amia*, *Atractosteus*, *Lepisosteus*) have a higher amount of estuarine or marine influence in their feeding [144]. It is also possible that the seasonally variable diet of *Lontra*, consuming mostly fish in summer and crustaceans/bivalves in winter, is responsible for its more divergent *δ*^13^C [145], as benthic bivalves often have considerably more negative carbon isotope compositions relative to littoral and pelagic/limnetic feeding taxa [138,142]. Carbon isotope compositions for bioapatite tissues from the sampled gar taxa are also of interest, as they record a higher degree of inter-tissue (i.e. signal in tooth enamel versus bone structural carbonate versus scale ganoine) variability than seen in other sampled taxa (electronic supplementary material, S1, tables S6 & S7 and figures S1 & S2). A possible explanation for this a high degree of variation in *δ*^13^C of bioapatite tissues in *Lepisoteus* and *Atractosteus* relates to their natural history. A study of seasonal habitat use in *Lepisosteus oculatus* from the Atchafalaya River (and surrounding lakes and streams) found considerable variation in home range size/shape [146]. Variation was particularly high during the annual spring flood pulse, at which time home ranges commonly expanded to include inundated floodplain terrain [146]. This variable habitat use during flooding could conceivably result in greater environmental variability in resources sources due to the mixing of freshwater aquatic with terrestrial and even marine sources, and consequently produce the greater variation obtained here for gar isotope data.

Resource mixing of the form suggested for gar may also account for some of the intra-species variability and overall isotopic range overlap seen in the Atchafalaya dataset. Just as this hypothesized mixing of organic carbon, sediment and even individual aquatic taxa (such as gar) may occur during annual flood-induced inundations, terrestrial taxa feeding across these landscapes may be consuming resources that are less distinct in isotopic composition than is the case in other environments. As well, living in annually flooded, often water-fragmented habitats may result in more mixed-feeding diets for taxa in these communities, deriving food resources from a mix of terrestrial and aquatic sources, rather than more specialized behaviours. An important consideration here is that without the observed natural history data available for this extant system (electronic supplementary material, table S2), the patterns in these *δ*^13^C data would be even more difficult to interpret. It is also possible that some component of the isotopic distributions of taxa sampled in these analyses may be influenced by other factors, such as species-specific ontogenetic dietary niche shifts [147,148], body size [149] or physiology [150], and further investigations into these questions may be warranted. Nevertheless, these results suggest that in coastal floodplain forest systems, considerable isotopic overlap between taxa, particularly where mean isotopic values are not significantly different, may obfuscate ecological and niche-use differences present in the sampled communities.

### 4.3. Oxygen isotopes and temperature-estimation

The overall pattern of oxygen isotope data from bioapatite is similar to the carbon isotope data in that a high degree of overlap exists among taxa (figure 2), which as noted above may be related to canopy effects, diet and humidity tolerance in some taxa. Surface water ‘temperatures’ were calculated from mean bioapatite oxygen isotope data (figure 3), and compared both among taxa and with the annual range of temperatures recorded directly from the Atchafalaya River. While some apparent patterns exist in the calculated temperatures, such as values for numerous aquatic or semi-aquatic taxa (e.g. *Lepisosteus* sp., *A. spatula*, *A. mississippiensis*, *M. coypus*, *L. canadensis*, *S. aquaticus*) all being within 1°C of one another, and the oxygen isotope compositions of these taxa being similar to unpublished USGS records of Atchafalaya River oxygen isotope data (–7.2 to –3.7‰, seasonally, with an average of –5.8‰, *sensu* Kendall, reported in Wagner [151]), this was not completely consistent. Some terrestrial taxa also had calculated temperatures very similar to those aquatic taxa, while certain aquatic taxa (e.g. *A. calva*) had much lower calculated temperatures. The reason for the difference in *δ*^18^O and calculated temperatures for *Amia* compared to other ectothermic fish such as *Lepisosteus* and *Atractosteus* is not known. It may represent a partial marine or estuarine signal in the gar taxa, as marine *δ*^18^O is typically higher than that of freshwater (due to factors such as precipitation, surface water, unless evaporated in areas of poor circulation) [21] and gar are known to frequent both freshwater and estuarine/marine settings [152]. *Amia* provides just one example of the many taxon-specific sources of variability that may exist. Behavioural factors (e.g. basking or shade-seeking in reptiles [65]), proportion of water obtained by drinking versus dietary sources (i.e. water contained in leaves and other vegetation), different tolerances to relative humidity conditions, and seasonal/annual changes in food availability may influence aspects of life history in these specific organisms that general oxygen isotope-temperature equations may not adequately take into account [5]. Similarly, it is also possible that some variability in the bioapatite oxygen isotope compositions, and corresponding temperature calculations, between sampled taxa may reflect differences in the seasonal timing of growth or remodelling of these tissues. Given that these data represent mean values from multiple individuals of different ages, they may not all correspond to the temperature/environmental conditions from the same temporal period. In addition to simply sampling individuals of different ages, taxonomic issues could also complicate this variability. For example, enamel oxygen isotope compositions of mammals would preserve a signal from an early ontogenetic stage (as permanent teeth in mammals form relatively early and are not replaced [133]), whereas those from fish or reptiles would be replaced on a regular basis throughout life and are located away from the body core (potentially leading to different formational temperatures) [65]. Bone bioapatite would presumably reflect more of an average oxygen isotope environmental signal given its longer remodelling time [134], though its oxygen isotope/temperature signal would also probably be influenced by ontogenetic and seasonal/temporal environmental differences between individuals and taxa. These factors may produce relatively small changes in oxygen isotope composition of a species, or may cause greater divergence in oxygen isotope composition when comparing one species to others, such as the case of *Odocoileus* in this dataset. The particularly high *δ*^18^O of *Odocoileus* compared to the other taxa may relate to it being a large-bodied folivore. It primarily consumes leaf water rather than surface water; leaf water is typically enriched in ^18^O relative to surface water by transpiration and greatly affected by humidity [4,20,49,111]. This higher oxygen isotope composition consequently impacts the calculated ‘temperature’ values from the bioapatite of this organism.

The uncertainties and associated variability present in the calculated temperatures derived from oxygen isotopes in vertebrate bioapatite in the Atchafalaya dataset present some possible problems for the use of such methods in palaeontological contexts. This variation, as noted above, may be the result of multiple factors influencing the preserved oxygen isotope composition, and potentially interfering with the oxygen isotope–temperature relationship. This suggests that alternative methods of isotope temperature reconstruction, such as the measurement of clumped isotopes [153], be used to confirm the utility of this proxy in palaeontological settings prior to widespread use. Despite these caveats, however, there may still be some cause for optimism in using oxygen isotope data from vertebrate bioapatite. Despite the variability in the oxygen isotope data here, the calculated mean temperatures for each taxon are within the range of temperatures recorded annually in the Atchafalaya River, and much more interestingly, the combined taxon mean temperature estimate (calculated from the proposed two-part endotherm-ectotherm method modified from Fricke & Wing [25]) of 21.9°C is within 2°C of the measured annual temperature (20.3°C) and 1°C of the measured annual median temperature (21.1°C) (figure 3 and table 4). The result of this combined taxon calculation approach suggests it may be a viable alternative to the variability of the single taxon methods, by providing an average ‘temperature’ estimate that is relatively free of the ‘noise’ present in individual taxon calculations (building on the two-taxon approach originally proposed by Fricke & Wing [25]). This ‘noise’ may be representative of different life histories and/or environmental preferences in the individual taxa, which in turn may bias the estimated ‘temperature’ calculated from their respective bioapatite oxygen. A wide sampling of taxa in a given system, representing a sufficiently wide breadth of life histories and relative environmental preferences may sufficiently cancel out the putative biases introduced by any individual taxon. If this is the case, then the modified method may be useful as an alternative to more intensive methods, such as clumped isotope measurements, in performing palaeo-temperature reconstructions, and may offer a way to minimize the impact of other factors affecting oxygen isotope composition (e.g. salinity, leaf water versus drinking water, evaporation etc.), depending on the specific taxa sampled and their proportion of the total sample. However, additional testing may be required, particularly against other methods of temperature estimation, to verify the efficacy of this modified multi-taxic method.

Although the relatively high degree of variability and overlap seen in the Atchafalaya results may appear somewhat at odds with other vertebrate isotope ecology studies [1,50,53,83], it is more typical of C_3_-dominated systems [61,154], and is consistent with the overlapping dietary and behavioural information available through observational natural history data of the taxa sampled from this system (electronic supplementary material, table S2). The isotopic results from the Atchafalaya system suggest a complex and considerable degree of resource mixing and aquatic–terrestrial interchange among taxa, and relative lack of resource ‘specialists’. If strong niche partitioning exists in this system, it may not involve variables/dimensions that can be easily detected via the isotopes analysed here. It is also possible given the degree of isotopic overlap that this system is currently unsaturated in terms of ecological resource use, allowing multiple taxa to utilize similar resources without extensive or exclusionary competition [155–158]. These results provide us with a rare example of a vertebrate community to ecosystem-scale sampling of isotopic distributions in a subtropical coastal floodplain forest environment. This case study thus provides us fundamental insight into the complex inter-relationships of the constituent taxa and an isotopic baseline for future comparisons in similar systems.

### 4.4. Implications and recommendations for isotopic studies of Mesozoic systems

As noted in the discussion of the isotope ecology of the ARB data themselves, one particularly important implication for interpreting isotopic data from Mesozoic systems is the degree to which ecological or dietary relationships can be interpreted in the presence of broad isotopic overlap between species. If overlap is considerable, and if there is not significant separation in the mean values between species, then drawing direct conclusions regarding the fine-scale ecological patterns present in such a system may not be possible (barring additional data that further contextualizes the results).

The relative relationships of bioapatite carbon and oxygen isotope distributions of taxa presented in this study (figures 2*b* and 3), particularly among groups like crocodilians (*Alligator*), ‘holostean’ fish (*Amia*, *Lepisosteus*, *Atractosteus*) and metatherians (*Didelphis*), should be useful for future direct comparisons with Mesozoic systems, given the presence of many morphologically, ecologically and/or phylogenetically similar taxa in such assemblages [45,70,159–162]. The environmental [41] and taxonomic [46] similarities between this study area and much of the Mesozoic terrestrial record (particularly in the Cretaceous) highlight the utility of the ARB dataset as a useful analogue comparator for Mesozoic system. In addition to these factors, the broad range of represented body sizes within both the endotherm–ectotherm and terrestrial–aquatic sample groups should further enhance the utility of the Atchafalaya dataset in facilitating comparisons with ancient and modern coastal floodplain forest ecosystems.

Another consideration from these data is their utility in constraining interpretations of isotopic results and informing on several sources of error/variation, or causes for caution, in isotopic studies of Mesozoic (and other pre-Cenozoic) systems. In several isotopic studies of Mesozoic dinosaurs, samples of gar ganoine have been used to facilitate intra- and inter-specific comparisons of herbivorous dinosaur enamel stable isotope data sampled from single sites or across formations [21,23,24]. While these comparisons may be of some use (such as in identifying shared relative offsets between taxa in different sites as a relative indicator of original biological signal preservation [23]), the use of gar (or any single taxon) as a consistent point of comparison [21,23] may be hampered by situations in which the isotopic compositions of the selected taxon vary considerably between sampling locations [21], a concern supported by the high variability observed in both *δ*^13^C and *δ*^18^O within and among enamel, bone and ganoine tissues from gar in this study, relative to other sampled taxa (figures 2 and 3; electronic supplementary material, figures S1 and S2). A possible method to control for some of sources of error and variability in pre-Cenozoic community studies is to sample a larger number of taxa, and to sample as many specimens as possible from a single locality and stratigraphic horizon. This should provide a reasonable approximation of the range of isotopic compositions in such a system, allow general ecological trends/patterns to be observed, and permit multiple temperature estimators (spanning the range of annual temperature variation) to be calculated. Additionally, our proposed modified two-part temperature estimate method, with sufficient taxonomic sampling, appears to allow for a surprisingly accurate approximation of mean annual water temperature to be captured. Given that the precise factors affecting species-level oxygen isotope composition may not be known when sampling fossil taxa, this method may mitigate some of that uncertainty. We would also recommend that additional methods for temperature estimation be employed to confirm these results.

Nitrogen isotope results in the Atchafalaya dataset, derived from keratin, are not particularly applicable to most pre-Cenozoic fossil stable isotope analyses due to a presumed lack of appropriate soft tissue preservation. In our view, previous research examining nitrogen isotope distributions (from high molecular weight compounds) in Mesozoic vertebrate communities [28] produced *δ*^15^N values that did not show the expected increase with increasing trophic level (albeit some suggestions to the contrary in that study), but instead exhibited a high degree of trophic level mixing (i.e. some secondary consumers with greater ^15^N than primary consumers, but also multiple secondary consumers with ^15^N considerably lower than any co-occurring primary consumer). Based on our results, it seems likely that those inconsistencies are the result of methodological issues, contamination or diagenetic alteration, or because nitrogen isotopes do not preserve in fossils of such considerable age. While evidence of original organic preservation in pre-Cenozoic fossils remains controversial [28,163–172], promising recent studies on the subject [173–175] suggest that further research on nitrogen isotope variation in these fossil systems is warranted.

A number of studies using carbon and/or oxygen isotope data have made ecological, environmental or physiological inferences about Mesozoic vertebrate communities and the taxa within them [21,23,28,33–35,159,176,177]. Our results should allow for both new research expanding on these foundational Mesozoic investigations, as well as the re-evaluation of previous hypotheses in the light of this new comparative framework. For example, Amiot *et al*. [159] performed palaeoenvironmental reconstructions and compared carbon and oxygen isotope compositions measured from multiple dinosaurs and reptiles sampled from six localities from the Cretaceous of East Asia, inferring evidence of niche partitioning of food resources. Three of their six sampled sites were compared statistically to assess for significant differences between carbon and oxygen isotope compositions in the sampled dinosaurs, with significant differences detected between taxa in two of the sites (table 3 in [159]). What is of particular interest in these sites with respect to the results from the Atchafalaya dataset is that fossil sites in Amiot *et al.* [159] with greater overlap between taxa possess wet/humid sub-tropical palaeoenvironments (not dissimilar from the ARB [71]), whereas those with greater apparent partitioning are reconstructed as cool temperate forests or warm temperate woodlands. It is conceivable that additional sampling in these sites, combined with comparisons with the Atchafalaya and other extant systems could expand upon the ecological patterns identified thus far and provide insight into resource-use and community structure across environmental gradients.

Similar to the previous example, Fricke & Pearson [21] compared carbon and oxygen isotope data from hadrosaurs and ceratopsians and concluded that micro-habitat niche-partitioning existed between them, with hadrosaurs suggested to live in floodplain forests and feed on plants of the forest canopy, and ceratopsians suggested to shift (over time) from a preference for plants in more open coastally influenced settings towards a preference for understorey plants in more closed canopy settings. Their results were based on the combined analysis of data from five vertebrate microfossil bonebeds (microsites), with statistical comparisons between hadrosaur and ceratopsian isotopic ranges within each site, as well as hadrosaur–hadrosaur and ceratopsian–ceratopsian ranges between larger multi-site groups. Statistically, hadrosaur and ceratopsian carbon isotope compositions were significantly different in 3/5 of sites, though this dropped to 1/5 of sites if the statistical analyses were performed on all samples from each site, as in two sites some hadrosaur teeth were removed from the final analyses due to concerns they represented misidentified ceratopsian teeth (due to plotting isotopically more similarly to ceratopsians rather than hadrosaurs, per Appendix 3 of [21]). Similarly, oxygen isotope compositions were identified as significantly different between hadrosaurs and ceratopsians in 1/5 of sites [21]. Though hadrosaurs and ceratopsians could often not be distinguished statistically, ceratopsians were found to be statistically distinct in carbon and oxygen isotope ranges from other ceratopsians when analysing among the larger multi-site groups, representing a possible temporal shift in diet or habitat-use in ceratopsians [21]. By comparison, in the Atchafalaya dataset, mean carbon and oxygen isotope compositions from bioapatite among herbivores such as *Sciurus*, *Myocastor* and *Sylvilagus* are approximately 2.0–4.5‰ and approximately 0.5–2.5‰ from one another, respectively (similar to hadrosaurs and ceratopsians), with broadly overlapping total isotopic ranges (figure 2). All three of these species were sampled from the same region, across a mixture of relatively closed to open canopy settings, and it is only through independent observational natural history records (electronic supplementary material, table S2) that differences in their microhabitat and dietary preferences can be readily identified. As the ceratopsians and hadrosaurs display inter-specific overlap in their isotopic ranges of a similar degree to those found between taxa in the Atchafalaya dataset, and that the identification of microhabitat niche-partitioning, if present, is often subtle in the Atchafalaya dataset (despite the advantage of possessing independent knowledge of those patterns from observational natural history data), a re-examination of the hypothesis of niche-partitioning through microhabitat preferences between herbivorous dinosaurs, or at least the degree of interpretive resolution possible from those data, may be warranted.

As a final example, a number of studies have identified that stable carbon isotope compositions in dinosaurs are much higher than would be expected for an extant vertebrate in a C_3_-based system [23,34]. Several explanations were suggested to explain this phenomenon, including differences in environmental/isotopic baseline [34], differences in consumed plant species or various stresses on those plants [21,34] or physiological or other factors resulting in dinosaurs possessing higher magnitude TEFs than seen in most extant vertebrates [21,23,34,159]. Interestingly, the carbon isotope compositions measured in Ostrom *et al.* [28] do not show this offset, though as noted above, the way in which those data were measured was atypical and thus may not be directly comparable. If combined with diverse multi-taxic isotopic sampling of dinosaurs and non-dinosaurs from a Mesozoic site, the Atchafalaya dataset could be integral in further testing these alternative hypotheses, as it provides an analogue system to be used as a comparative isotopic baseline, and includes multiple taxa from a wide variety of diets, physiologies and autecologies.

An important consideration for future interpretations of deep-time vertebrate isotope distributions is that extant analogue datasets can provide a useful isotopic baseline and point of comparison to facilitate the testing of previously hypothesized ecological patterns in Mesozoic vertebrate isotope studies, and may be of particular value given that they facilitate independent confirmation of results (via comparison with observational natural history data) and lack the confounding concern of diagenetic alteration that accompanies any investigation of ancient stable isotopes [4]. From the high degree of isotopic overlap among taxa in the Atchafalaya system, we might predict both considerable aquatic–terrestrial resource intermixing, and possible ecological undersaturation (in terms of resource use and the severity of competitive exclusion) in similar systems found in the Mesozoic [155–157]. As well, it is probably the case that niche partitioning in these coastal floodplain forest systems is occurring in ways not easily detectable via carbon and oxygen isotope proxies (such as through feeding-height stratification [178]). This combination of factors may account for some of the diversity present in assemblages of co-occurring herbivorous and faunivorous dinosaurs found in settings such as the Late Cretaceous of western North America [179–182].

## 5. Conclusion

This study provides the first broad-scale vertebrate isotope ecology case study with multi-taxic, multi-tissue and multi-isotope sampling from a modern C_3_-dominated coastal floodplain forest system. Measured ranges of *δ*^13^C (from keratin and bioapatite tissues) and *δ*^18^O (from both carbonate and total oxygen sampling of bioapatite) were found to have considerable overlaps among individuals and taxa, suggesting a high degree of resource mixing, terrestrial–aquatic interchange, and a possible lack of ecological saturation in this system. Even with well-established natural history data on ecological variables for our ARB taxa, such as trophic habits and microhabitat use, the subtleties involved in identifying these patterns in our isotopic data highlight the difficulties involved in detecting and interpreting ecological patterns in a complex C_3_-based system. It stands to reason that for a palaeoecological system where the description of niches often requires significant inference (particularly among non-avian dinosaurs, for which there is no exact extant analogue), using isotopic data to describe fine-resolution details of niche partitioning should be done conservatively. Consequently, though carbon isotope data can provide some meaningful ecological data in these systems, we also recommend that novel stable isotope systems be considered in future investigations of trophic and niche structure in C_3_ ecosystems. Our proposed multi-taxic modification to the two-part endotherm–ectotherm equation provides a surprisingly accurate method of temperature estimation, and may be of considerable use in palaeoenvironmental analyses. This research provides a fundamental comparative baseline for assessing isotopic variation and testing ecological hypotheses in future studies of analogous or near-analogous Mesozoic systems. Comparisons of this nature should assist in constraining predictions and limiting the degree to which palaeoecological-proxy data may be over-interpreted.

## Supplementary Material

Supplementary Information 1

## Supplementary Material

Supplementary Figure 1

## Supplementary Material

Supplementary Figure 2

## Supplementary Material

Supplementary Table 1

## Supplementary Material

Supplementary Table 2

## Supplementary Material

Supplementary Table 3

## Supplementary Material

Supplementary Table 4

## Supplementary Material

Supplementary Table 5

## Supplementary Material

Supplementary Table 6

## Supplementary Material

Supplementary Table 7

## Supplementary Material

Supplementary Table 8

## Data accessibility

The datasets and additional analyses supporting this article are contained within the electronic supplementary material.
