# Supplementary material for: Stable isotopic characterization of a coastal floodplain forest community: a case study for isotopic reconstruction of Mesozoic vertebrate assemblages
Source: R Soc Open Sci. 2019 Feb 20;6(2):181210. doi: 10.1098/rsos.181210 (PMC6408390; doi:10.1098/rsos.181210)
Supplement: Supplementary Information 1 [file rsos181210supp1.docx]

Supplementary Information 1 – Methodological tests and tissue-level analyses

Introduction:

Further to the ecological objectives stated in the main body of this manuscript, the following sections detail methodological tests and tissue-level examinations performed to ensure the reliability and comparability of the isotopic data analyzed for this paper. Specifically, this supplement details methodological tests performed to confirm the broad comparability of isotopic data derived from different tissues of similar structure (e.g. hair and claw keratin, or bone and enamel bioapatite) for use in the primary ecological comparisons of this study. In addition, literature-sourced trophic enrichment factors (TEFs) are compared to isotopic tissue differences measured in the sampled taxa, and used in conjunction with measured keratin and bioapatite isotopic data to determine reasonable TEF estimates for bioapatite in sampled reptiles where previously only keratin TEFs were known.

The following hypotheses are here tested:

1. Tissues formed from similar materials (e.g. hair and claw, or bone and tooth) will not have significantly different isotopic ranges for a given taxonomic group, and/or will have highly correlated isotopic compositions in individual specimens with such paired tissues.
2. Isotopic fractionation between soft and hard tissues will match literature predictions, and the calculated diet isotopic compositions from hard and soft tissue will be similar for a given species.
3. For cases where direct isotopic fractionation data are unknown (e.g. crocodilian bioapatite) for one tissue but known for another (keratin), isotopic offsets between keratin and bioapatite (*Δ*_bioapatite-keratin_) compositions in reptiles sampled here (i.e. *Alligator*) will be consistent with previous studies examining *Δ*_bioapatite-diet_ or *Δ*_keratin-diet_ in other reptile taxa.

Material and Methods:

Sample collection and laboratory procedures are as described in the main body of the manuscript. Data analysis and statistical tests were performed using the R programming language and contained core packages (1), along with the ‘lmodel2’ package (2). Shapiro-Wilk normality tests were performed on all analysis datasets to assess the distribution of the data, and to determine if parametric statistical tests were appropriate. For each data subset requiring statistical hypothesis testing, F-tests were performed to determine equal or unequal variance, followed by either two sample t-tests or Welch two sample t-tests, respectively (3). Unless otherwise stated, tissue-level comparisons refer to specific measurements taken from particular tissues of indicated individuals, individual-level comparisons refer to the mean values of all measurements taken for a particular specimen, and species-level comparisons refer to the mean value obtained for a given species through averaging all individual-level means for the species (with standard deviations and standard errors calculated from the individual means used to compute the overall species mean).

Comparisons were made examining the difference in stable carbon isotope data from surface-treated versus fully treated bone samples obtained using laser ablation or conventional powder analysis for all available specimens (N_specimens_ = 11, N_taxa_ = 5). Additional comparisons were made between bioapatite structural carbonate *δ*^18^O and bioapatite laser-produced total-oxygen (LPTO) *δ*^18^O to test if differences between these values in the same organism match expected carbonate-phosphate-LPTO offsets (such as 7.5‰ (4), or 8.7‰ from (5)). This was performed to confirm the suitability of our conversions of non-phosphate/phosphate-equivalent measurements to phosphate-equivalent values (to facilitate our later ecological comparisons). This comparison was made for all available samples with measurements from both bone structural carbonate and bone phosphate (N_specimens_ = 22, N_taxa_ = 7), with intensive sub-sampling given to two specimens, AWR-135 (*Alligator mississippiensis*) and AWR-06 (*Didelphis virginiana*).

In order to assess the internal comparability of keratin and bioapatite data for stable carbon, oxygen, and nitrogen isotope ratios, ranged major axis (RMA) regressions were calculated for individuals possessing: hair keratin and claw keratin samples, and/or bone bioapatite and tooth enamel bioapatite samples (2, 3). Further sub-sample regression analyses were performed within the sampled fish taxa, as their data included additional tissues (e.g. ganoine) that were not present in any other group. In each case the slopes, r^2^ values, and p values were recorded. Additionally, the differences in each mean isotope ratio and the effect of differing forms of pre-treatment were recorded for each tissue for each individual specimen (Supplementary Table 5).

Results

Data Summary

Stable carbon, oxygen, and nitrogen isotope data for each sampled tissue from each specimen analyzed are summarized in Table 1 and Supplementary Table 5. Individual stable isotopic measurements are reported in Supplementary Table 4. Shapiro-Wilk tests confirmed the normality of each stable isotope dataset (keratin *δ*^15^N, W = 0.98, p = 0.04; keratin *δ*^13^C, W = 0.94, p = 3.12x10^-5^; bioapatite *δ*^13^C, W = 0.98, p = 2.19x10^-5^; bioapatite *δ*^18^O, W = 0.99, p = 0.007).

Methodological Tests & Tissue Comparisons

Statistical tests of tissue comparisons are summarized in Supplementary Table 6 (regression data) and Supplementary Table 7 (F- and t-tests). Variation in stable isotope measurements taken from individual specimen tissues were typically less than 1‰, with average intra-tissue variability (indicated by standard deviation, see Supplementary Table 5) being 0.28‰ for nitrogen, 0.56‰ for carbon, and 0.93‰ for oxygen. Variation in δ^13^C between surface pre-treated (S) and additionally/fully pre-treated (Tr) bone samples was dependent on analytical procedure, with mean *Δ*_S-Tr_ equal to 3.78‰±2.27‰ for laser ablated bone samples and 0.09‰±0.97‰ for conventional powder analyzed bone samples. Average oxygen isotope fractionation between structural carbonate (obtained through conventional powder analyses) and laser-produced total-oxygen (obtained through laser ablation analyses) measured in bone samples was determined to be 7.03‰±2.11‰, similar to expected literature values (4, 6, 7). Statistical testing confirmed significant differences in structural carbonate and LPTO *δ*^18^O in more intensive repeat analyses of bone samples in both AWR-135 (F = 1.14, p = 0.895; t-test with equal variance, t = -27.42, p=0.0001) and AWR-06 (F = 71.31, p=0.15; t-test with equal variance, t = -15.90, p = 0.004) (Supplementary Figure 1A, Supplementary Table 7). From these comparisons (and those in the aforementioned literature), carbonate-phosphate (~ –6‰) and LPTO-phosphate (~ –1‰) discriminations could be determined for converting all oxygen isotope data to phosphate-equivalent compositions for community comparisons.

No statistically significant differences were detected in *δ*^13^C between bone structural carbonate and tooth enamel for the dataset as a whole (F = 0.76, p = 0.420; t-test with equal variance, t = 1.67, p=0.099), for reptiles (F = 0.75, p = 0.792; t-test with equal variance, t = -1.54, p = 0.162), for omnivorous/faunivorous mammals (F = 0.53, p = 0.279; t-test with equal variance, t = 1.45, p = 0.161), or for herbivorous mammals (F = 0.77, p = 0.836; t-test with equal variance, t = -0.88, p = 0.412), though significant differences were found for *δ*^13^C between bone structural carbonate and tooth enamel for fish (F = 0.53, p = 0.281; t-test with equal variance, t = 2.58, p = 0.017) (Supplementary Table 7). All subset comparisons of *δ*^13^C between bone structural carbonate and tooth enamel produced slopes close to one (fish: slope = 1.44, r^2^ = 0.14, p = 0.127; reptile: slope = 1.14, r^2^ = 0.87, p = 0.007; herbivorous mammal: slope = 1.1, r^2^ = 0.47, p=0.133; omnivorous/faunivorous mammal: slope = 1.39, r^2^=0.78, p=0.0009) when plotted using RMA regression, though the low r^2^ value for fish indicates that relatively little of those data are explained through this relationship (Supplementary Figure 1B, Supplementary Table 6). Significant differences exist in *δ*^18^O between bone LPTO and tooth enamel LPTO for reptiles (F=1.00, p=0.997; t-test with equal variance, t = 4.42, p = 0.002), but not for fish (F = 0.94, p=0.954; t-test with equal variance, t = 0.70, p = 0.5), omnivorous/faunivorous mammals (F = 0.83, p= 0.809; t-test with equal variance, t = -0.13, p = 0.901), herbivorous mammals (F = 0.2, p = 0.336; t-test with equal variance, t = 0.88, p = 0.43), or the dataset taken as a whole (F = 1.60, p=0.301; t-test with equal variance, t=1.92, p = 0.062) (Supplementary Table 7). Subset comparisons of *δ*^18^O between bone phosphate and tooth enamel phosphate produced highly varying slopes (fish: slope = 0.64, r^2^ = 0.11, p = 0.269; reptile: slope = -0.99, r^2^ = 0.75, p = 0.038; herbivorous mammal: slope = -0.42, r^2^ = 0.09, p = 0.51; omnivorous/faunivorous mammal: slope = 1.15, r^2^ = 0.11, p = 0.233) when plotted using RMA regression (Supplementary Figure 1C, Supplementary Table 6).

Statistical comparisons of *δ*^15^N from hair and claw keratin of the same individuals were not significant for the dataset as a whole (F = 0.60, p = 0.431; t-test with equal variance, t = -0.43, p = 0.670), for omnivorous/faunivorous mammals (F = 0.97, p = 0.983; t-test with equal variance, t = -0.19, p = 0.859), or for herbivorous mammals (F = 0.57, p = 0.480; t-test with equal variance, t = -0.83, p = 0.423) (Supplementary Table 7). Subset comparisons of *δ*^15^N between hair and claw keratin produced slopes close to one (herbivorous mammal: slope = 1.02, r^2^ = 1.02, p = 0.044; omnivorous/faunivorous mammal: slope = 1.32, r^2^ = 0.89, p = 0.002) when plotted using RMA regression (Supplementary Figure 1D, Supplementary Table 6). Statistical comparisons of *δ*^13^C ratios for keratin tissues (hair vs. claw) were not significant for the dataset as a whole (F = 0.96, p = 0.941; t-test with equal variance, t = 0.11, p = 0.911), for omnivorous/faunivorous mammals (F = 0.78, p = 0.842; t-test with equal variance, t = 0.33, p = 0.756), or for herbivorous mammals (F = 0.99, p = 0.988; t-test with equal variance, t = -0.16, p = 0.872) (Supplementary Table 7). Subset comparisons of *δ*^13^C between hair and claw keratin produced slopes close to one (herbivorous mammal: slope = 1.13, r^2^ = 0.91, p=0.032; omnivorous/faunivorous mammal: slope=1.01, r^2^ = 0.69, p = 0.007) when plotted using RMA regression (Supplementary Figure 1E, Supplementary Table 6). Statistical comparisons of *δ*^18^O ratios for hair and claw keratin were not significant for the dataset as a whole (F = 10.11, p = 0.002; Welch t-test, t = 1.25, p = 0.236) or for herbivorous mammals (F = 4.44, p = 0.128; t-test with equal variance, t = -0.99, p = 0.344), but were significant for omnivorous/faunivorous mammals (F = 1.82, p = 0.636; t-test with equal variance, t=4.13, p = 0.006) (Supplementary Table 7). Subset comparisons of *δ*^18^O between hair and claw keratin produced varying slopes (herbivorous mammal: slope = 0.74, r^2^ = 0.82, p = 0.047; omnivorous/faunivorous mammal: slope = -0.089, r^2^ = 0.0006, p = 0.46) when plotted using RMA regression (Supplementary Figure 1F, Supplementary Table 6).

Given the consistently low r^2^ values of fish in the above comparisons, and their possession of a third bioapatite tissue (ganoine), the fish (and particularly gar) isotopic data were investigated further. Regressions of fish bone and tooth enamel *δ*^13^C produced varying slopes (*Amia*: slope = 2.23, r^2^ = 0.75, p = 0.125; *Atractosteus*: slope = 1.49, r^2^ = 0.62, p=0.075; *Lepisosteus*: slope = -1.6, r^2^ = 0.06, p = 0.503) (Supplementary Figure 2A, Supplementary Table 6). Statistical comparisons of *δ*^13^C for bone and tooth enamel did not find significant differences for *Amia* (F = 0.20, p = 0.23; t-test with equal variance, t = 0.06, p = 0.960) or *Lepisosteus* (F = 0.38, p = 0.436; t-test with equal variance, t = 1.97, p = 0.09), but did for *Atractosteus* (F = 0.38, p = 0.436; t-test with equal variance, t = 1.97, p = 0.09) and the gar data taken together (F = 0.81, p=0.77; t-test with equal variance, t = 3.20, p = 0.005) (Supplementary Table 7). Regressions of gar ganoine and tooth enamel *δ*^13^C produced differing slopes (*Atractosteus*: slope=1.31, r^2^=0.35, p=0.072; *Lepisosteus*: slope=-0.69, r^2^=0.95, p=0.032) (Supplementary Figure 2B, Supplementary Table 6). Statistical comparisons of *δ*^13^C for ganoine and tooth enamel did not find significant differences for *Atractosteus* (F=1.84, p=0.569; t-test with equal variance, t=-1.35, p=0.210) or *Lepisosteus* (F=0.49, p=0.569; t-test with equal variance, t=-1.63, p=0.15), but did when gar data were analyzed together (F=1.21, p=0.790; t-test with equal variance, t=-2.18, p=0.045) (Supplementary Table 7). Regressions of gar ganoine and bone *δ*^13^C produced more similar slopes (*Atractosteus*: slope=0.91, r^2^=0.41, p=0.049; *Lepisosteus*: slope=0.42, r^2^=0.19, p=0.326) (Supplementary Figure 2C, Supplementary Table 6) than were calculated when comparing ganoine to tooth enamel. Statistical comparisons of *δ*^13^C ratios for ganoine and bone did not find significant differences in *Atractosteus* (F=0.77, p=0.810; t-test with equal variance, t=1.76, p=0.120), *Lepisosteus* (F=0.18, p=0.193; t-test with equal variance, t=-0.35, p=0.74), or when gar data were analyzed together (F=98, p=0.979; t-test with equal variance, t=1.08, p=0.298) (Supplementary Table 7). Regressions of gar ganoine LPTO and tooth enamel LPTO *δ*^18^O produced differing slopes (*Atractosteus*: slope=-0.52, r^2^=0.05, p=0.297; *Lepisosteus*: slope=0.46, r^2^=0.87, p=0.231) (Supplementary Figure 2D, Supplementary Table 6). Statistical comparisons of *δ*^18^O of ganoine LPTO and tooth enamel LPTO did not detect significant differences in *Atractosteus* (F=0.31, p=0.279; t-test with equal variance, t=-1.15, p=0.290), but did in *Lepisosteus* (F=0.21, p=0.232; t-test with equal variance, t=-3.15, p=0.02) and when gar data were analyzed together (F=0.29, p=0.009; t-test with equal variance, t=-2.88, p=0.01) (Supplementary Table 7).

Statistical comparisons were also made for mean stable carbon and oxygen isotope ratios between keratin and bioapatite in a subset of the total sampled taxa to determine the average isotopic separation between these tissues and overall range of variation for each species (Supplementary Table 7). A difference in *δ*^13^C between bioapatite and keratin tissues is apparent in *Alligator* (F=2.32, p=0.963; t-test with equal variance, t=7.66, p=8.89x10^-8^), *Didelphis* (F=2.46, p=0.087; t-test with equal variance, t=11.96, p=6.43x10^-14^), and *Sylvilagus* (F=0.59, p=0.403; t-test with equal variance, t=6.62, p=2.46x10^-6^). Significant differences also existed between the mean *δ*^18^O ratios of bioapatite and keratin tissues in *Alligator* (F=75.54, p=0.181; t-test with equal variance, t=5.09, p=3.78x10^-5^), *Didelphis* (F=0.54, p=0.2; t-test with equal variance, t=3.74, p=0.0007), and *Sylvilagus* (F=43.85, p=4.21x10^-5^; Welch t-test, t=6.62, p=1.75x10^-5^).

When adjusting for multiple comparisons in t-test results (using Bonferroni correction with α=0.05 and N_tests_=40), the threshold for a significant p-value became ~0.001. Under this adjusted p-value, all significant relationships in Supplementary Table 7 are no longer detected as significant.

4. Discussion

4.1 Tissue-, Specimen-, & Species-level Variation

Comparisons made at various levels in the sampled data confirmed that, in general, isotopic variation for a given element (e.g., C, N, O) was low for individual tissues within a specimen, moderate between tissues of a specimen, and moderate to high between specimens and species. There are several possible explanations for this variability.

Some of variability in the isotopic data may be of analytical origin, as different/repeated sampling of the same specimen results in similar means but higher variability when using laser ablation vs bulk powder methods, suggesting that bulk sampling homogenizes some natural variability that can be detected when performing smaller-scale laser ablation sampling (Supplementary Tables 2 and 5). Differences in sample treatment prior to bulk analysis had a relatively limited effect on the resulting *δ*^13^C, with the difference between the surface-treated samples and the samples given additional pre-treatment being on average 0.09‰ for bulk powder samples. The higher offsets between surface-treated and additionally-treated samples subjected to laser heating is consistent with the higher variability in laser ablation results in general (Supplementary Table 5). This higher variability is consistent with some previous studies (4), and may reflect the more targeted nature of laser sampling. Sampling at the 10’s of *µ*m scale, as is possible using laser heating, can reveal temporal variations in the isotopic signal (e.g. summer-winter signals, diet shifts) not detectable in by analysis of bulk powders. Differences in oxygen isotope data obtained from carbonate and LPTO of the same samples have an average fractionation of 7.03‰, broadly consistent with literature expectations (4). The low isotopic variability of most repeated samples for particular specimens for all analyzed isotopes, along with the matching of literature expectations for differences expected among sampling methodologies, suggests that non-analytical processes (e.g. ecological, physiological, ontogenetic, *post-mortem* alteration) may account for much of the measured variability.

Testing of isotopic data for similar tissues confirmed a general absence of statistical difference (particularly in the case of Bonferroni-corrected results) and in many cases the results from similar tissues are tightly correlated when sampled from the same individual (Supplementary Figure 1, Tables 5 & 6). Some exceptions exist, however, particularly among the sampled gar taxa. LPTO data for reptile bone and tooth enamel were also statistically different, though strongly correlated. This may be related to the different temporal scales recorded in these two tissues, as tooth replacement in *Alligator* has been shown to occur on the order of ~80-120 days (slowing with age) (8), whereas skeletal bone replacement takes multiple years (9). At these differing scales, it is possible that tooth enamel in these samples recorded seasonal or short-term differences (i.e. winter vs. summer, differing food availability, degree of oxygen obtained via drinking vs. consumed tissue) that are otherwise obscured or averaged out in the bone data (10). Among the sampled gar taxa, there did not appear to be a consistent pattern in isotopic signal when comparing bioapatite tissues (tooth enamel, bone, and ganoine) of given individuals or species (Supplementary Figures 1 & 2, Supplementary Tables 2, 6, and 7). Similar to some of the difference detected in *Alligator*, it is possible that this is issue is temporal (i.e. seasonal difference in resource availability/composition, differing formation times of sampled tissues). It is also possible that the distribution, development, and turnover of different bioapatite tissues may be more complex in actinopterygians (like gar) than in tetrapods, as gar possess both enamel and enameloid on their teeth (enamel over the shaft of the tooth and enameloid at the tip), and have bony ganoine-coated scales (homologous with enamel, but not enameloid) (11, 12). This complexity may also lead to an increased rate of accidental mixing of tissues during sampling (i.e. bone from ganoine scales, enameloid instead of enamel from teeth). Without better data for the developmental and re-modelling patterns in these tissues, as well as gar tissue-diet and tissue-drinking water isotopic spacings, the exact cause of this increased variability cannot be explicitly determined. Lastly, the reason for the discordant oxygen isotope signals from claw and hair keratin in omnivorous/faunivorous mammals is not known, and the mechanisms by which oxygen (as well as other isotopes) is incorporated into the soft tissues of omnivores remains somewhat controversial (10, 13-18). For this reason (as well as limited available data), keratin oxygen results were not used for the broader community comparisons in this study.

As noted above, as a result of the Bonferroni-corrections for multiple comparisons few of the above comparisons remained significant. This outcome may reflect either accurate correction of type I errors, or introduction of type II errors, given the conservative nature of the Bonferroni-correction (19). Given the latter possibility, the relationships among these data, and hypotheses as to their possible causes, are still discussed. Most major tetrapod groups have been the subjects of one or more laboratory experiments to determine expected tissue-diet (TEF) and/or tissue-drinking water spacing, with this being particularly true in mammals (Supplementary Table 3) (20). Comparisons between keratin and bioapatite *δ*^13^C for sampled mammalian taxa (Supplementary Table 5), particularly the more intensively subsampled *Didelphis* and *Sylvilagus* (Supplementary Table 7), have significantly different means and match expected literature offsets (within the expected range of variability arising from temporal or other factors, as discussed above) (Supplementary Table 3). In the case of *Alligator*, *Δ*^13^C_keratin-diet_ have been determined from laboratory experiments (21, 22), though it appears that only distantly related reptiles have empirically determined *Δ*^13^C_bioapatite-keratin_ (23). Based on those literature data, an expected *Δ*^13^C_bioapatite-keratin_ for *Alligator* is ~7.5‰, and the observed *Δ*^13^C_bioapatite-keratin_ here appears to be ~9‰ (Supplementary Tables 3 & 5). Given that empirically determined tissue fractionation factors are known to vary by as much as 4‰ in a single species under relatively controlled conditions (24), and that the factors leading to fractionation differences in a single individual/species are still not fully understood (13, 14, 16, 17, 25), the predicted and observed *Δ*^13^C_bioapatite-keratin_ for *Alligator* can be considered largely congruent.

Conclusions:

Methodological tests confirm that in general the isotopic data for tissues formed from similar materials in the same taxa are congruent, and their tissue-diet and tissue-tissue TEFs match literature predictions. Measured oxygen isotope compositions from carbonate and LPTO sources display offsets consistent with literature expectations. Isotopic variance is unexpectedly high in gar taxa for different sampled bioapatite tissues, and controlled feeding & growth experiments are needed to interpret the results more fully. Values of *Δ*^13^C_bioapatite-keratin_ determined for *Alligator* match predictions derived from *Δ*^13^C_keratin-diet_ and *Δ*^13^C_bioapatite-diet_ in other reptile taxa, allowing for reasonable estimates of *Δ*^13^C_keratin-diet_ and *Δ*^13^C_bioapatite-diet_ to be computed for the purposes of this study without direct observations from laboratory diet experiments.

References:

1. R-Development-Core-Team. R: a language and environment for statistical computing. 2.12.0 ed. Vienna, Austria: R Foundation for Statistical Computing; 2015.

2. Legendre P. Model II Regression. 1.6-3 ed: CRAN; 2008.

3. Legendre P, Legendre LF. Numerical ecology: Elsevier; 2012.

4. Larson TE, Longstaffe FJ. Deciphering seasonal variations in the diet and drinking water of modern White-Tailed deer by in situ analysis of osteons in cortical bone. Journal of Geophysical Research: Biogeosciences. 2007;112(G4):n/a-n/a.

5. Bryant JD, Koch PL, Froelich PN, Showers WJ, Genna BJ. Oxygen isotope partitioning between phosphate and carbonate in mammalian apatite. Geochimica et Cosmochimica Acta. 1996;60(24):5145-8.

6. Cerling TE, Sharp ZD. Stable carbon and oxygen isotope analysis of fossil tooth enamel using laser ablation. Palaeogeogr Palaeocl. 1996;126:173-86.

7. Sharp Z, Cerling T. A laser GC-IRMS technique for in situ stable isotope analyses of carbonates and phosphates. Geochimica et Cosmochimica Acta. 1996;60(15):2909-16.

8. Erickson GM. Daily deposition of dentine in juvenile Alligator and assessment of tooth replacement rates using incremental line counts. J Morphol. 1996;228(2):189-94.

9. Hall BK. Bones and cartilage: developmental and evolutionary skeletal biology: Academic Press; 2005.

10. Crowley BE, Melin AD, Yeakel JD, Dominy NJ. Do Oxygen Isotope Values in Collagen Reflect the Ecology and Physiology of Neotropical Mammals? Frontiers in Ecology and Evolution. 2015;3:1:12.

11. Berkovitz BK, Shellis RP. The Teeth of Non-mammalian Vertebrates: Academic Press; 2016.

12. Braasch I, Gehrke AR, Smith JJ, Kawasaki K, Manousaki T, Pasquier J, et al. The spotted gar genome illuminates vertebrate evolution and facilitates human-teleost comparisons. Nat Genet. 2016;48(4):427-37.

13. Perga M-E, Grey J. Laboratory measures of isotope discrimination factors: comments on Caut, Angulo & Courchamp (2008, 2009). Journal of Applied Ecology. 2010;47(4):942-7.

14. Caut S, Angulo E, Courchamp F, Figuerola J. Trophic experiments to estimate isotope discrimination factors. Journal of Applied Ecology. 2010;47(4):948-54.

15. del Rio CM, Wolf N, Carleton SA, Gannes LZ. Isotopic ecology ten years after a call for more laboratory experiments. Biol Rev Camb Philos Soc. 2009;84(1):91-111.

16. Caut S, Angulo E, Courchamp F. Variation in discrimination factors (Δ15N and Δ13C): the effect of diet isotopic values and applications for diet reconstruction. Journal of Applied Ecology. 2009;46(2):443-53.

17. Caut S, Angulo E, Courchamp F. Discrimination factors (Δ15N and Δ13C) in an omnivorous consumer: effect of diet isotopic ratio. Functional Ecology. 2008;22(2):255-63.

18. Kurle CM, Koch PL, Tershy BR, Croll DA. The effects of sex, tissue type, and dietary components on stable isotope discrimination factors (Δ13C and Δ15N) in mammalian omnivores. Isotopes in Environmental and Health Studies. 2014;50(3):307-21.

19. Rothman KJ. No adjustments are needed for multiple comparisons. Epidemiology. 1990:43-6.

20. Kohn MJ, Cerling TE. Stable isotope compositions of biological apatite. In: Kohn MJ, Rakovan J, Hughes JM, editors. Phosphates Geochemical, Geobiological, and Materials Importance. Reviews in Mineralogy and Geochemistry. 48. Washington, D. C.: Mineralogical Society of America; 2002. p. 455-88.

21. Rosenblatt AE, Heithaus MR. Slow isotope turnover rates and low discrimination values in the American alligator: implications for interpretation of ectotherm stable isotope data. Physiol Biochem Zool. 2013;86(1):137-48.

22. Whiting ET, Steadman DW, Krigbaum J. Paleoecology of Miocene crocodylians in Florida: Insights from stable isotope analysis. Palaeogeography, Palaeoclimatology, Palaeoecology. 2016;451:23-34.

23. Biasatti DM. Stable carbon isotopic profiles of sea turtle humeri: implications for ecology and physiology. Palaeogeography, Palaeoclimatology, Palaeoecology. 2004;206(3-4):203-16.

24. Sare DTJ, Millar JS, Longstaffe FJ. Tracing dietary protein in red-backed voles (Clethrionomys gapperi) using stable isotopes of nitrogen and carbon. Canadian Journal of Zoology. 2005;83(5):717-25.

25. Auerswald K, Wittmer MHOM, Zazzo A, Schäufele R, Schnyder H. Biases in the analysis of stable isotope discrimination in food webs. Journal of Applied Ecology. 2010;47(4):936-41.
