# Supplementary figures and images for: Stable isotopic characterization of a coastal floodplain forest community: a case study for isotopic reconstruction of Mesozoic vertebrate assemblages
Source: R Soc Open Sci. 2019 Feb 20;6(2):181210. doi: 10.1098/rsos.181210 (PMC6408390; doi:10.1098/rsos.181210)

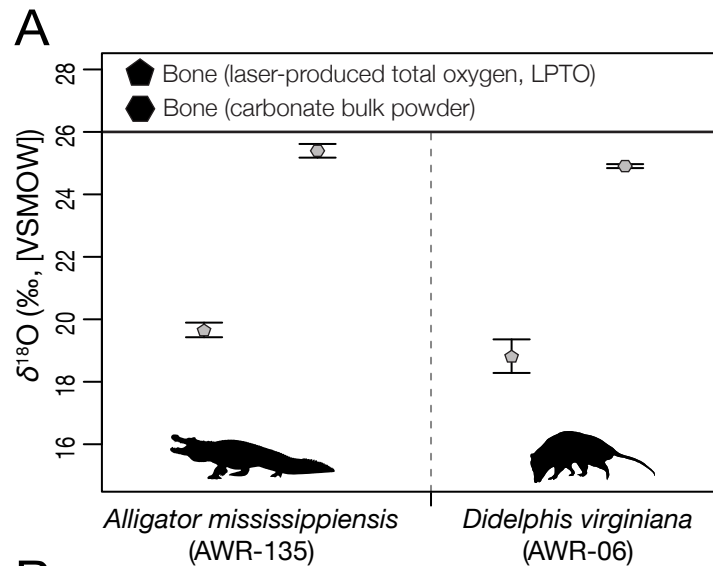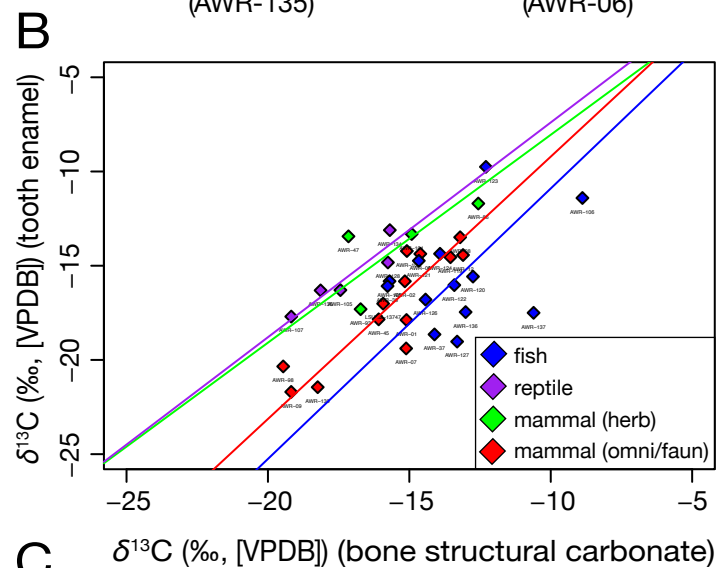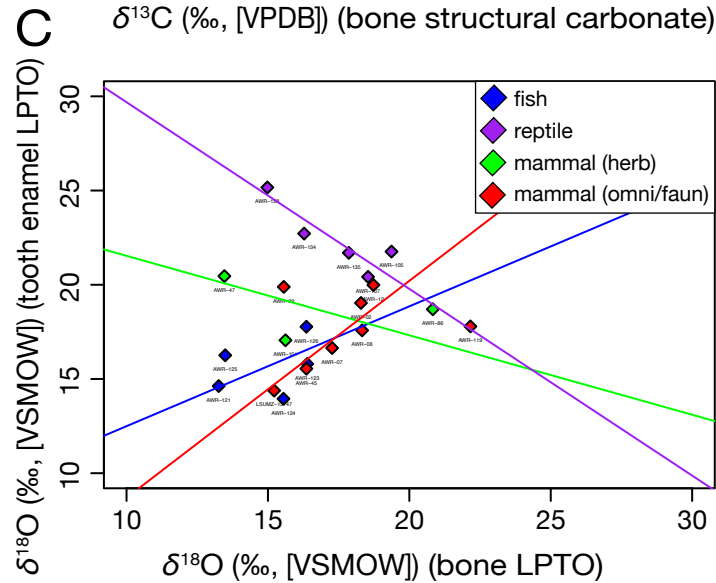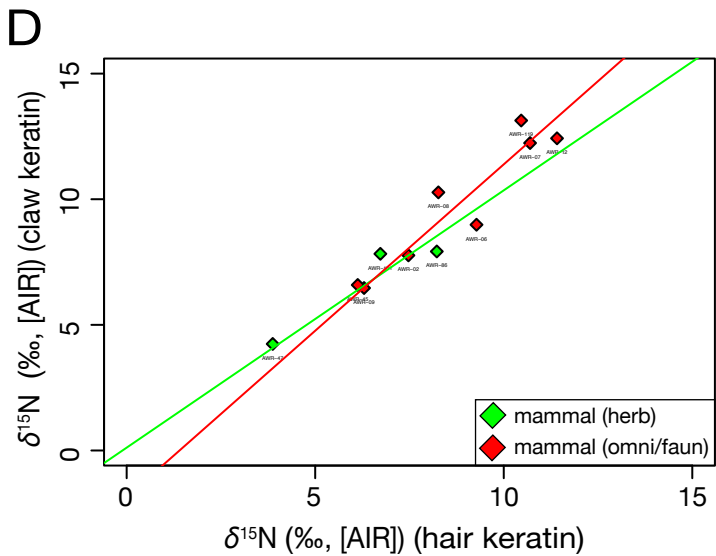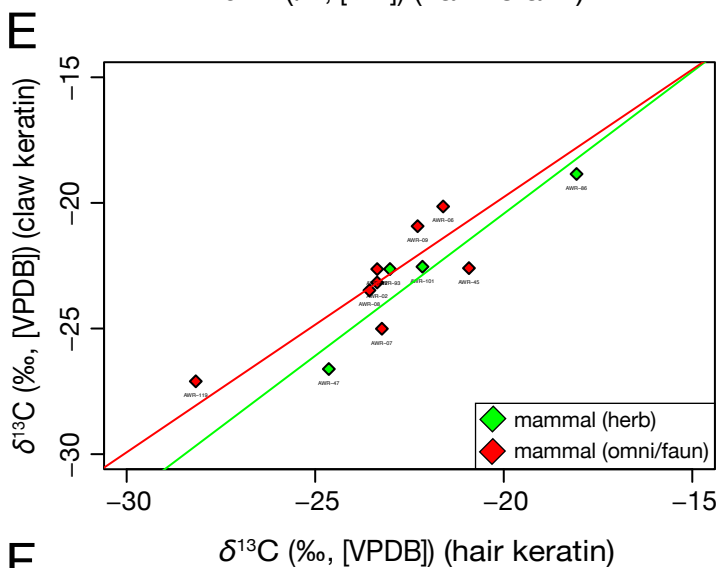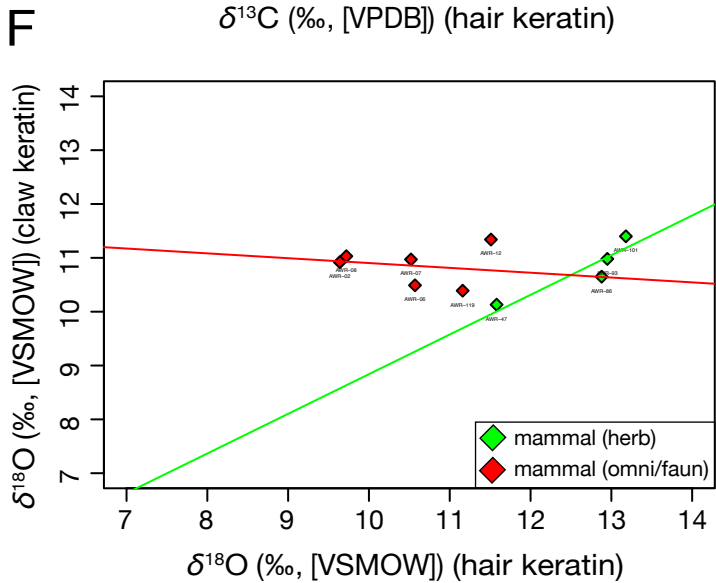

Supplement: Supplementary Figure 1 [file rsos181210supp2.pdf]

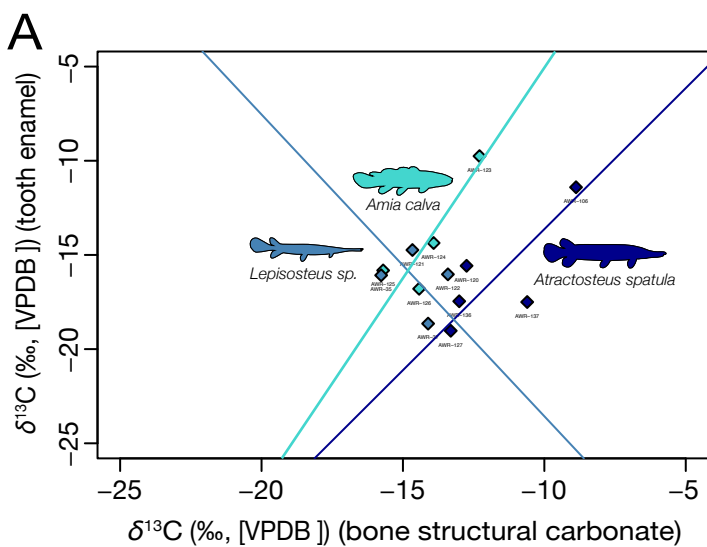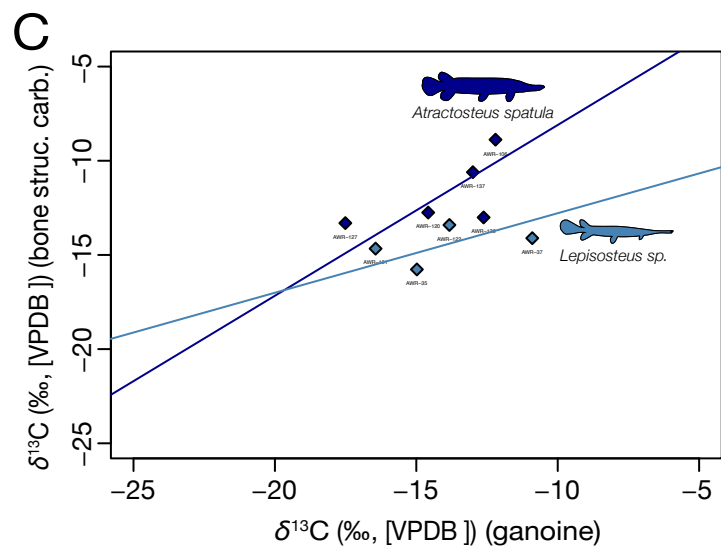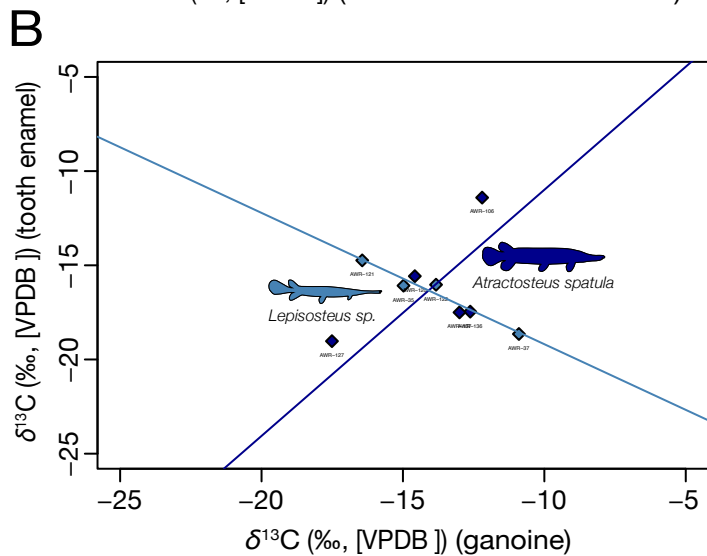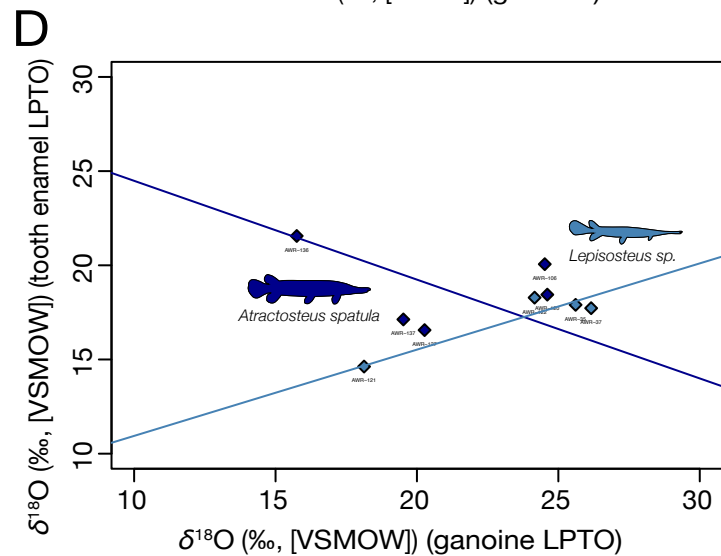

Supplement: Supplementary Figure 2 [file rsos181210supp3.pdf]
